# A Programmable Wafer-scale Chiroptical Heterostructure of Twisted Aligned Carbon Nanotubes and Phase Change Materials

**DOI:** 10.1038/s41467-025-59600-w

**Published:** 2025-05-14

**Authors:** Jichao Fan, Ruiyang Chen, Minhan Lou, Haoyu Xie, Nina Hong, Benjamin Hillam, Jacques Doumani, Yingheng Tang, Weilu Gao

**Affiliations:** 1 Department of Electrical and Computer Engineering, The University of Utah, Salt Lake City, UT USA; 2 J.A. Woollam Co., Inc., Lincoln, NE USA; 3 Department of Electrical and Computer Engineering, Rice University, Houston, TX USA; 4 Elmore Family School of Electrical and Computer Engineering, Purdue University, West Lafayette, IN USA

**Keywords:** Carbon nanotubes and fullerenes, Carbon nanotubes and fullerenes, Materials for optics, Optical materials

## Abstract

The ability to design and dynamically control chiroptical responses in solid-state matter at a wafer scale enables new opportunities in various areas. Here, we present a full stack of computer-aided designs and experimental implementations of a dynamically programmable, unified, scalable chiroptical heterostructure containing wafer-scale twisted aligned one-dimensional carbon nanotubes and non-volatile phase change materials. We develop a software infrastructure based on high-performance machine learning frameworks, including differentiable programming and derivative-free optimization, to efficiently optimize the tunability of both reciprocal and nonreciprocal circular dichroism responses, which are experimentally validated. Further, we demonstrate the heterostructure scalability regarding stacking layers and the dual roles of aligned carbon nanotubes - the layer to produce chiroptical responses and the Joule heating electrode to electrically program phase change materials. This heterostructure platform is versatile and expandable to a library of one-dimensional nanomaterials, phase change materials, and electro-optic materials for exploring novel chiral phenomena and photonic and optoelectronic devices.

## Abstract (web summary)

Tunable chiroptical structures are of extensive interest. Here, the authors show designs and experimental implementations of a programmable, scalable chiroptical heterostructure containing twisted aligned carbon nanotubes and phase change materials.

## Introduction

Manipulating chiroptical responses in solid-state materials and their photonic and optoelectronic devices has enabled various applications, including sensing^1,2^, imaging^3^, neuromorphic computing^4^, and light-driven synthesis^5^. In particular, the interaction of circularly polarized light with quantum materials plays a crucial role on quantum photonic applications^6,7^. Chiroptical responses, such as circular dichroism (CD) that is the differential attenuation between the left- and right-handed circularly polarized (LCP and RCP) light, have two prominent contributions with distinct features^8,9^. The first is an intrinsic isotropic reciprocal response due to molecular or structure-induced chirality, which is invariant upon sample orientation. The second is a linear-anisotropy-induced nonreciprocal response, leading to opposite handednesses from opposite sides of the same structure. Leveraging and dynamically manipulating both contributions of chiroptical responses can enrich functionalities, improve efficiencies, and reduce costs of chiral photonic and optoelectronic devices^10^.

Engineered metamaterials hybridized with electrically, thermally, or mechanically controllable materials or architectures can enable dynamic programmability of strong chiroptical responses^11–15^. For example, phase change materials (PCMs) can be fast programmed between crystalline and amorphous phases with a substantial dielectric function modulation in broadband spectral ranges and their material phases are preserved with zero energy consumption after removing external stimulus^16,17^. Although these favorable properties have been utilized to construct tunable metamaterials^18–21^, there is limited demonstration of controlling chiroptical responses. In addition, the top-down manufacturing of tunable artificial symmetry-breaking structures is sophisticated and challenging to scale up^22,23^. Further, their design based on full-wave simulations is accurate but time-consuming and challenging for fast design and lately demonstrated machine learning (ML) approaches offer fast but approximate solutions^24^. Recently, twisted stacks of bottom-up self-assembled aligned 1D nanomaterials, such as carbon nanotubes (CNTs), have emerged as a high-performance chiroptical material platform and demonstrated the highest CD response compared to all other material and metamaterial platforms in the deep ultraviolet range^25^. The large CD response originates from the strong room-temperature quantum-confinement-induced optical resonances in CNTs compared to natural molecules and self-assembled conventional metallic and dielectric materials without quantum-relevant properties^26^. Further, these resonances can span a broadband range from the ultraviolet to the infrared. However, no platform can utilize, efficiently and accurately design, and dynamically program both reciprocal and nonreciprocal CD responses.

Here, we show a fully programmable, unified, wafer-scale chiroptical heterostructure of twisted aligned 1D nanomaterials and non-volatile chalcogenide PCMs, whose reciprocal and nonreciprocal CD responses can be designed through ML-powered frameworks and electrically programmed. The demonstrated 1D nanomaterial and PCM are CNT and germanium-antimony-tellurium (Ge_2_Sb_2_Te_5_ or GST), respectively. We implement simulation software based on ML frameworks, including graphics processing unit (GPU)-powered differentiable programming enabled by gradient-based backpropagation algorithm and derivative-free Bayesian optimization, to efficiently design and optimize the tunable ranges of heterostructure reciprocal and nonreciprocal CD responses using experimentally obtained material optical constants. We further experimentally implement designed heterostructures through wafer-scale low-cost self-assembly of aligned CNTs and wafer-scale deposition of GST and dielectric films, and observe strong dynamic tunability. In particular, we demonstrate tunable nonreciprocal CD responses, which display the sign or polarity reversal of measured CD signals in a broadband visible range when opposite sides of the same heterostructure are probed. All measured spectra agree with simulations. In addition, we demonstrate that not only the lateral dimension of the heterostructure is at wafer scale, but also the vertical dimension is scalable with a large number of stacking layers to enhance CD responses. Moreover, we show that aligned CNTs in the heterostructure play dual roles – the active material to produce CD responses and the Joule heating electrode material to electrically program GST phases. The demonstrated heterostructure architecture is versatile and can be extended to incorporate a wide range of 1D nanomaterials, such as transition metal dichalcogenides nanotubes^27^, boron nitride nanotubes^28^, nanotube heterostructures^29^, and encapsulated CNTs with 1D atomic chains^30^, and different PCMs and electro-optic materials^31–33^, such as antimony sulfide (Sb_2_S_3_), antimony selenide (Sb_2_Se_3_), lithium niobate (LiNO_3_) and electro-optic organic materials. This versatile chiroptical platform provides opportunities for novel chiral photonic and optoelectronic devices, such as chiral quantum light emitters^34^, and provides a new platform for exploring optical and non-optical chiral phenomena, such as chirality-induced spin selectivity effects^35^.

## Results

### Programmable heterostructure architecture

**Fig. 1:**
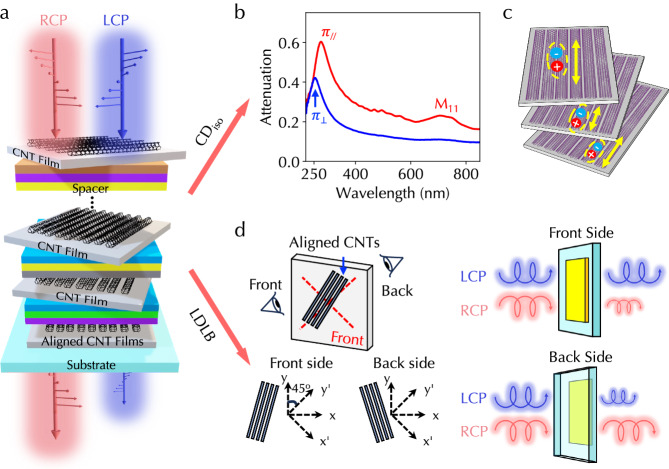
Programmable heterostructure architecture and chiroptical responses. **a** Illustration of programmable chiroptical heterostructure containing twisted aligned CNTs and isotropic PCMs and dielectrics. **b** Linear-polarization-dependent absorption spectra of an aligned CNT film prepared using the shaking-assisted vacuum filtration process. The red (blue) line for parallel (perpendicular) polarization. **c** Isotropic reciprocal CD_iso_ response in the heterostructure originating from the helical coupling of anisotropic 1D excitons in aligned CNTs. **d** Nonreciprocal LDLB response in the heterostructure originating from the interference of linear anisotropy, which displays opposite handednesses from the front and back sides of the same sample. Source data are provided as a Source Data file.

Figure 1a schematically illustrates the programmable heterostructure architecture containing multiple layers of anisotropic aligned CNTs with different orientations and other isotropic materials, including PCMs and dielectrics. The quantum confinement along CNT circumferences induces the formation of subbands and excitonic interband transitions across subbands. These transitions span broadband spectral ranges and are dependent on CNT atomic structures^36^. Further, the 1D geometry of CNTs leads to anisotropic excitonic electric dipoles and optical absorption under the excitation of linearly polarized light. We used a shaking-assisted vacuum filtration (SAVF) process to prepare aligned CNT films and measured their linear-polarization-dependent absorption spectra. The linear shaking occurring during the vacuum filtration dictated the alignment direction of obtained aligned CNT films (Methods and Supplementary Fig. 1). The deterministic control of CNT alignment direction in the SAVF process is advantageous over conventional vacuum filtration without direction control and facilitates the scalable stacking of multiple layers in the heterostructure. As shown in Fig. 1b, when the polarization of incident light is parallel to the CNT axis, strong absorption occurs through M_11_ transition in CNTs peaked at 730 nm and the *M* point transition (labeled ‘*π*_∥_’) at 282 nm^37^. The absorption resonance wavelength is dependent on CNT diameters and atomic structures^38^. In contrast, a different *M* point transition (labeled ‘*π*_⊥_’) occurs at 255 nm due to optical selection rules^39^ and the M_11_ transition is suppressed because of the depolarization effect^40^. The helical twist of anisotropic excitonic dipoles and electromagnetic coupling between them, essentially corresponding to the physical picture of helically coupled electrical dipoles in microscopic chiral molecules^41^, produce reciprocal CD response (CD_iso_); see Fig. 1c. The CD_iso_ response is intrinsic, isotropic, and independent of sample orientation. The incorporation of PCMs and other isotropic materials in the heterostructure can dynamically control the electromagnetic coupling between dipoles in aligned CNTs and CD_iso_ response.

In addition, the other component of observed CD response originates from the interference of linear dichroism (LD) and linear birefringence (LB) of aligned CNTs (Fig. 1d). This CD response is called LDLB response and is expressed as 0.5(${{{{\rm{LD}}}}}^{{\prime} }$LB  − LD${{{{\rm{LB}}}}}^{{\prime} }$), where LD and LB are defined in arbitrarily chosen *x*–*y* axes and ${{{{\rm{LD}}}}}^{{\prime} }$ and ${{{{\rm{LB}}}}}^{{\prime} }$ are defined along bisectors of *x*-*y* axes with a  + 45° rotation. Note that the LDLB response is a genuine chiroptical CD response instead of an effect for linearly polarized light^8,9^. When looking from the front or back side of the aligned CNT plane (i.e., flipping the sample), aligned CNTs become mirrored with LD and LB unaltered and ${{{{\rm{LD}}}}}^{{\prime} }$ and ${{{{\rm{LB}}}}}^{{\prime} }$ signs flipped. Hence, the sign of the LDLB response is also flipped and called nonreciprocal CD^8,9^. If the LDLB response is much larger than isotropic CD_iso_, the measured CD spectra can display opposite signs when measuring from opposite sample sides (Fig. 1d). The LDLB response is non-zero only when the main axes of LD and LB are not aligned with each other; otherwise two terms in the LDLB expression are always canceled. However, the optical axes of maximum attenuation for LD and phase delay for LB of CNTs are both along the CNT axis and minimum ones are both perpendicular to the CNT axis, meaning LD and LB quantities are aligned. This suggests the LDLB response be zero in CNT-only architectures. The incorporation of PCMs and other isotropic materials in the heterostructure can create and control multiple reflections between layers. Because LD (LB) is related to the imaginary (real) part of the CNT refractive index, the multiple reflections inside the same heterostructure can have different influences on LD and LB and their main axes are decoupled and different from the CNT axis. Hence, the heterostructure not only can generate non-zero LDLB response but also can dynamically tune and optimize LDLB response.

### Simulation framework

**Fig. 2:**
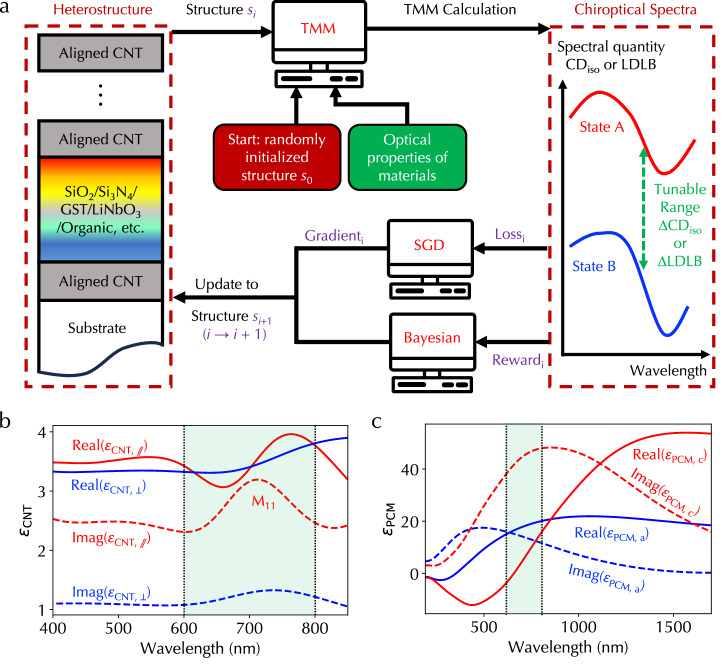
Simulation framework based on machine learning frameworks. **a** Flowchart of designing the chiroptical heterostructure to achieve prominent dynamic tunability ranges of CD_iso_ or LDLB (*Δ*CD_iso_ or *Δ*LDLB) through differentiable programming enabled by the stochastic gradient descent (SGD) algorithm through the backpropagation algorithm in PyTorch and derivative-free Bayesian optimization algorithm. The framework takes input from experimentally determined complex-valued dielectric functions of (**b**) an anisotropic aligned CNT film and (**c**) a sputtered isotropic GST film. In (**b**), the red (blue) solid line is the real part of the dielectric function of the aligned CNT film along (perpendicular to) the CNT axis. In (**c**), the red (blue) solid line is the real part of the dielectric function of the GST film under the crystalline (amorphous) phase. The dashed lines are imaginary parts in both (**b**) and (**c**). Source data are provided as a Source Data file.

Figure 2a illustrates our developed simulation framework to design heterostructures to achieve prominent tunability of CD_iso_ and LDLB responses. We developed a general 4 × 4 transfer matrix method (TMM) to obtain all transmission and reflection coefficients under different linear and circular polarizations (Methods and Supplementary Fig. 2). We calculated these coefficients under four configurations of sample rotation and flipping to obtain CD_iso_ and LDLB spectra (Methods and Supplementary Fig. 3). Since all fundamental operations in TMM are matrix multiplications, we implemented the TMM framework using the PyTorch framework so that all calculations were GPU-accelerated in parallel. The TMM input included the dielectric functions of anisotropic and isotropic materials and a set of heterostructure structural parameters *s*, such as the choice of materials, layer thicknesses, and rotation angles between aligned CNTs. Figure 2b, c display our experimentally determined complex-valued dielectric functions of an aligned CNT film through multi-curve fittings and a sputtered GST film under amorphous and crystalline phases through spectroscopic ellipsometry (Methods and Supplementary Fig. 4). Supplementary Fig. 5 also displays the complex-valued refractive index of GST under two phases. We selected the wavelength range of 600–800 nm covering the M_11_ transition of CNTs because of the relatively low optical loss of GST at least in one phase in this range and the limitation of the spectrometer measurement range (shaded green areas in Fig. 2b, c, and Supplementary Fig. 5). Note that in addition to GST, other PCMs and electro-optic materials, such as electro-optic LiNbO_3_ and organic materials, can also be incorporated into the framework for heterostructure design.

The design process started with a randomly initiated structure *s*_0_, which was input into the TMM to calculate CD_iso_ and LDLB responses when the dielectric functions of amorphous and crystalline GST were used. The difference of CD_iso_ (LDLB) spectra in the wavelength range of interest under two GST states, denoted as *Δ*CD_iso_ (*Δ*LDLB), was used to define optimization target functions. In principle, any quantity calculated from transmission and reflection coefficients can become target functions, such as the dissymmetry *g* factor defined as the ratio of CD_iso_ over the attenuation under unpolarized light and any functions of *Δ*CD_iso_ and *Δ*LDLB. We developed two different categories of optimization algorithms. The first is based on the stochastic gradient descent algorithm implemented using the PyTorch-supported backpropagation algorithm. Since the backpropagation algorithm aims to minimize a target loss function, the loss function was defined as the most negative absolute value of *Δ*CD_iso_ or *Δ*LDLB spectra. Based on the gradient calculated from the backpropagation algorithm, in *i*-th iteration, the set of structural parameters *s*_*i*_ was updated to *s*_*i*+1_ to decrease the loss function and equivalently to increase *Δ*CD_iso_ or *Δ*LDLB. The optimal heterostructure was obtained after multiple iterations. The second is the derivative-free Bayesian optimization algorithm. Note that although our developed PyTorch TMM solver is differentiable, the derivative-free algorithm can be broadly applicable even when gradients are not available. The target of the Bayesian optimization algorithm is to maximize the reward function. Hence, we defined the largest absolute value of *Δ*CD_iso_ or *Δ*LDLB spectra as the reward function. In each iteration, based on prior sets of structural parameters and reward functions, the algorithm determined the Bayesian posterior probability and a new set of structural parameters through sampling. After multiple iterations, the heterostructures with large *Δ*CD_iso_ or *Δ*LDLB were highly probably sampled.

### Experimental demonstration

**Fig. 3:**
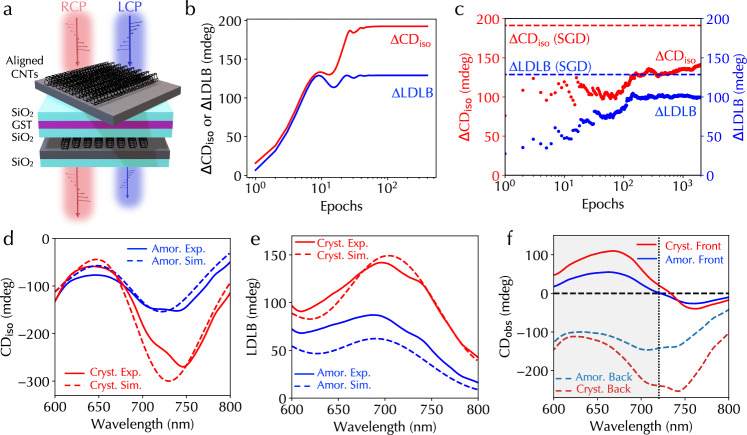
Simulation and experimental implementation of a programmable heterostructure with two layers of aligned CNT films. **a** Illustration of the specific heterostructure containing two layers of twisted aligned CNT films. Training curves of *Δ*CD_iso_ (red lines in (**b**) or dots in (**c**)) and *Δ*LDLB (blue lines in (**b**) or dots in (**c**)) using (**b**) differentiable programming and (**c**) derivative-free Bayesian optimization, respectively. Red (blue) dashed lines in (**c**) indicate optimized values for *Δ*CD_iso_ and *Δ*LDLB from (**b**). Measured (solid lines) and simulation (dashed lines) spectra of (**d**) CD_iso_ and (**e**) LDLB responses under GST crystalline (red lines) and amorphous (blue lines) phases, respectively. **f** Observed CD spectra measured from the front (solid lines) and back (dashed lines) sides of the same heterostructure under GST crystalline (red lines) and amorphous (blue lines) phases. The gray shaded area indicates the wavelength range showing the polarity reversal. Source data are provided as a Source Data file.

Figure 3a illustrates a specific heterostructure consisting of two layers of twisted aligned CNTs, two silicon oxide (SiO_2_) layers, and one GST layer between SiO_2_ layers to demonstrate the optimization using the simulation framework and experimental validation. The twisted angle between aligned CNTs and the thicknesses of SiO_2_ and GST layers were structural parameters to be optimized. The aligned CNT thickness was assumed to be a constant and benchmarked by comparing the ultraviolet attenuation with a standard sample (see Methods). Figure 3b, c display training curves using the differentiable backpropagation algorithm and derivative-free Bayesian optimization algorithm, respectively, for optimizing *Δ*CD_iso_ and *Δ*LDLB. For Bayesian optimization, Figure 3c shows the accumulated average of *Δ*CD_iso_ and *Δ*LDLB. In comparison, the average values from the Bayesian optimization are lower than those from the backpropagation algorithm because of relatively wide parameter ranges. If parameter ranges were narrower, the Bayesian optimization produced similar optimization values to the backpropagation algorithm (Supplementary Fig. 6a). The obtained twisted angle between two aligned CNT layers was  ~45° in both algorithms (Supplementary Fig. 6b, c), consistent with the optimized angle in CNT-only twisted stack^25^. A full list of optimized structural parameters is summarized in Supplementary Table 1. We further performed simultaneous optimizations of *Δ*CD_iso_ and *Δ*LDLB using the backpropagation algorithm by defining the loss function as a linear combination of *Δ*CD_iso_ and *Δ*LDLB (Methods and Supplementary Fig. 7). The results not only demonstrate the versatility of the optimization using arbitrary customized targets in our developed simulation framework but also reveal a trade-off of optimizing *Δ*CD_iso_ and *Δ*LDLB, leading to sub-optimal results compared to separate optimizations. Furthermore, the observed trade-off highlights the fundamental mechanism difference between CD_iso_ and LDLB chiroptical responses.

We fabricated optimized heterostructures by transferring SAVF-prepared aligned CNT films and sputter-depositing GST and SiO_2_ films on fused silica substrates, which are transparent in the measurement range of 200−900 nm (see Methods). Similar to simulations, we performed four CD measurements using a standard CD spectrometer with a compatible 3D-printed sample holder under four configurations of sample rotation and flipping to obtain CD_iso_ and LDLB responses (Methods and Supplementary Fig. 3). The as-deposited GST film was in the amorphous phase and was converted into the crystalline phase by heating using a hotplate (Supplementary Fig. 8 and Supplementary Movie 1). Figure 3d, e show measured CD_iso_ and LDLB spectra (solid lines) when the GST film was under amorphous and crystalline phases, showing agreement with the simulation spectra (dashed lines) using the developed simulation framework. Note that the difference between experiments and simulations is mainly because the aligned CNT films used for obtaining optical constants in Fig. 2b could be slightly different from the films in heterostructures due to slight manufacturing variations in the SAVF process. The maximum obtained *Δ*CD_iso_ was 123 mdeg at 750 nm wavelength and the maximum obtained *Δ*LDLB was 60 mdeg at 737 nm wavelength. Broadband CD_iso_ and LDLB spectra under two GST phases are shown in Supplementary Fig. 9a, b. As shown in Supplementary Fig. 9c, d, the measured average absorption spectra of LCP and RCP light of the heterostructures under two GST phases (solid lines) also showed agreement with simulations (dashed lines). Further, we designed and optimized the heterostructure for the tunable range of *g* factor using the backpropagation algorithm, and experimentally measured spectra also agreed with simulation spectra (Supplementary Fig. 10). This demonstrates the broad applicability of the simulation framework and experimental implementation of the heterostructure.

In particular, prominent LDLB responses were observed in Fig. 3e. For the same heterostructure, Fig. 3f and Supplementary Fig. 11 display measured CD spectra (CD_obs_) when the front and back sides of the heterostructure faced the direction of incident light (see Methods). We observed a clear sign or polarity reversal of CD_obs_ in a broadband wavelength range of 424–721 nm for the GST amorphous phase and a wavelength range of 421–735 nm for the crystalline phase. As mentioned before, the LDLB response originates from the difference between intensity attenuation and phase delay extrema due to the interference of multiple reflections in the heterostructure. To confirm this, we further employed the developed simulation framework to calculate linear-polarization-dependent transmitted intensity and the phase delay between transmitted light and input light at a specific wavelength (Supplementary Fig. 12a). The zero degree was defined when the orientation of the first aligned CNT layer and the polarization direction were the same. Supplementary Fig. 12b, c display the calculated normalized attenuation and phase delay as a function of polarization angle at 630 nm for two GST phases, showing clear shifts of extrema positions. While maximum phase delay positions occurred at 0° under both GST phases, maximum attenuation positions occurred at 16. 7° and 13. 3°, respectively. Note that these values are also different from the orientation of second-layer aligned CNTs, which is 45° under the coordination system defined in Supplementary Fig. 12a. We also experimentally measured attenuation at 630 nm of the heterostructure as a function of polarization angle, confirming the shift (Supplementary Fig. 12d).

The incorporation of the GST film creates multiple reflections among interfaces in the heterostructure. The tunable optical constants of the GST film regulate the amplitude and phase of reflected waves and control the electromagnetic interaction between twisted aligned CNTs, which is the origin of CD responses (Supplementary Fig. 13)^25^. Hence, general elliptical eigenmodes, CD_iso_, and LDLB responses are programmable. Note that the phase delay and optical loss gained when passing through the GST film are isotropic, modify eigenmodes in the same manner, and do not change CNT interaction (Supplementary Fig. 13b). Further, to understand the contributions of the change of the real and imaginary parts of optical constants of the GST film to *Δ*CD_iso_ and *Δ*LDLB, we performed simulations and optimizations by artificially assuming that only real or imaginary parts were tunable (Methods and Supplementary Fig. 14). CD_iso_ and LDLB spectra of optimized heterostructures in Fig. 3d, e were similar to those obtained using artificial GST films with only imaginary part tuning (Methods and Supplementary Figs. 15–18). Further, the optimized values obtained with imaginary-part-only-tuning GST films were larger than those obtained with real-part-only-tuning GST films, and *Δ*CD_iso_ and *Δ*LDLB values and spectra (Methods and Supplementary Figs. 19–21) were close to those in Fig. 3b, d, and e. These observations suggest that the tunable imaginary parts of the dielectric function or refractive index mainly contribute to *Δ*CD_iso_ and *Δ*LDLB. Note that the tunable imaginary parts of the dielectric function or refractive index lead to the change of both reflected and absorbed light in the GST film.

### Layer scalability

**Fig. 4:**
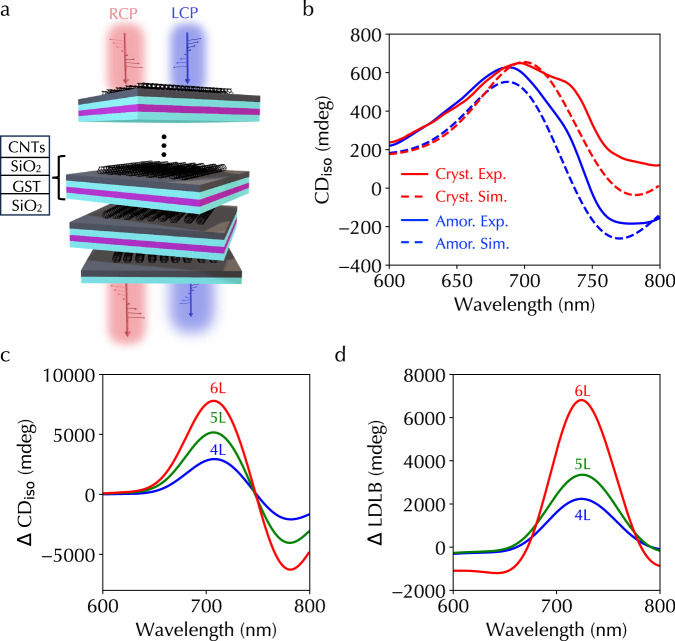
Layer scalability of chiroptical responses in the programmable heterostructure. **a** Illustration of heterostructures containing multiple CNTs-SiO_2_-GST-SiO_2_ units. **b** Experimentally measured (solid lines) and simulation (dashed lines) CD_iso_ spectra under GST amorphous (blue lines) and crystalline (red lines) phases, respectively. **c** Simulation *Δ*CD_iso_ and (**d**) *Δ*LDLB spectra for heterostructures containing four (blue lines), five (green lines), and six layers (red lines) of twisted aligned CNT films. Source data are provided as a Source Data file.

Further, we demonstrated that both the simulation framework and experimental implementation are scalable with respect to the number of stacking layers in the heterostructure. Specifically, we repeated CNTs-SiO_2_-GST-SiO_2_ configuration in the heterostructure to have three layers of aligned CNT and GST films following the same CNT transfer and film deposition processes (Fig. 4a). Figure 4b shows experimentally measured CD_iso_ spectra under two GST phases (solid lines), which agree with simulation CD_iso_ spectra (dashed lines). The maximum *Δ*CD_iso_ was 363 mdeg at 752 nm wavelength, which is nearly three times as large as that of the heterostructure with two aligned CNT films in Fig. 3d. In addition, we designed heterostructures containing four, five, and six layers of twisted aligned CNT films using the backpropagation algorithm to optimize *Δ*CD_iso_ and *Δ*LDLB. Note that the total number of layers in the heterostructure containing six aligned CNT films is 21. Figure 4c, d show their corresponding simulation spectra. The obtained maximum *Δ*CD_iso_ and *Δ*LDLB for the six-CNT-layer heterostructure were 7.8 deg and 6.8 deg, respectively. The CD_iso_ and LDLB spectra for four-, five-, and six-CNT-layer heterostructures under two GST phases are shown in Supplementary Figs. 22, 23, respectively.

The overall attenuation or insertion loss of the heterostructure also increases with the number of CNT and GST layers (Supplementary Fig. 24) and the detection limit in practical applications can restrict the maximum layer number. If the attenuation detection limit of 5 in standard CD spectrometers is used as a benchmark, the four-CNT-layer heterostructure is the upper scaling limit. Replacing GST with other low-loss PCMs, such as Ge_2_Sb_2_Se_4_Te (GSST), Sb_2_S_3_, and Sb_2_Se_3_^19,21,42^, can increase the upper limit. We designed heterostructures to optimize *Δ*CD_iso_ and *Δ*LDLB for various CNT layers by artificially assuming zero imaginary parts of the dielectric function or refractive index in the actual GST optical properties (Fig. 2c and Supplementary Fig. 5). Not only the upper scaling limit but also the maximum *Δ*CD_iso_ and *Δ*LDLB were more than double under the same detection limit benchmark (Methods and Supplementary Fig. 25), highlighting the benefits of utilizing low-loss PCMs in the chiroptical heterostructure. Further, these large obtained *Δ*CD_iso_ and *Δ*LDLB using zero-loss materials suggest the tunable absorbed light in GST is not the major contributor (Supplementary Fig. 13).

### Electrical tunability

**Fig. 5:**
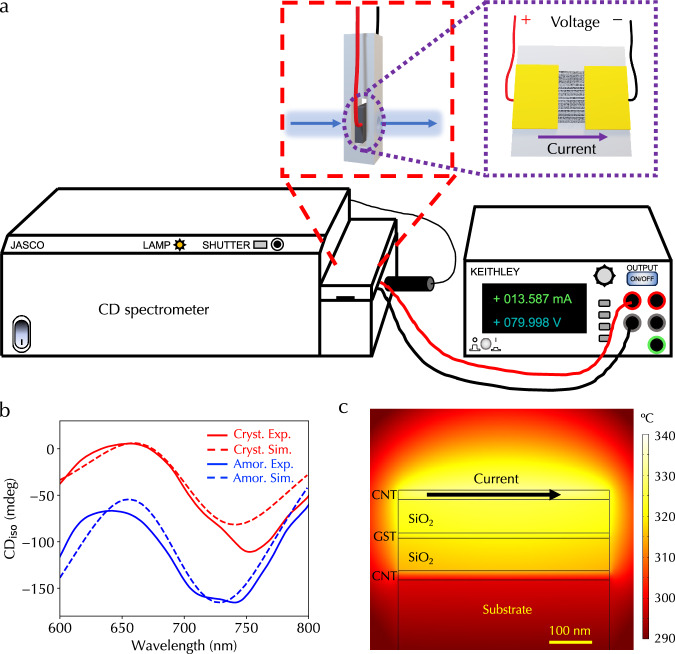
Electrical tunability of chiroptical responses in the heterostructure. **a** Illustration of in-situ programming of the heterostructure in the CD spectrometer. **b** Experimentally measured (solid lines) and simulation (dashed lines) CD_iso_ spectra under GST amorphous (blue lines) and crystalline (red lines) phases, respectively. **c** Spatial temperature distribution of a scaled-down heterostructure. Source data are provided as a Source Data file.

In addition to contributing to chiroptical responses through twisted stacks, the high thermal conductivity, low heat capacity, and reliable and high current carrying capacity of aligned CNTs^43^ make them an efficient Joule heating electrode for programming GST phases. Hence, the aligned CNT film in the heterostructure can play dual roles as both chiroptical and electrode materials. Figure 5a illustrates in-situ programming of the heterostructure in the CD spectrometer. Two electrodes and wires were in contact with the top layer of the aligned CNT film, with the current flowing along the alignment direction. The sample was loaded into the developed 3D-printed sample holder compatible with the spectrometer. We first measured CD spectra under four sample configurations, then applied voltages across the sample through a sourcemeter, and measured CD spectra again under four sample configurations. Figure 5b displays measured CD_iso_ spectra (solid lines), which agreed with simulation spectra (dashed lines) as well. The applied voltage and current for converting the GST film from amorphous to crystalline phases were  ~80 V and  ~13.6 mA, respectively. We further performed heat transfer simulations using the COMSOL Multiphysics software to estimate the temperature profile (see Methods). Figure 5c shows a spatial distribution of temperature in the heterostructure with scaled-down lateral dimension and delivered electrical energy from the top aligned CNT film. The achieved temperature inside the GST film can be  ~320 °C, which is much larger than the GST transition temperature from amorphous to crystalline phase (150 °C–200 °C), confirming the crystallization of GST film.

The switching from crystalline to amorphous phases, called the re-amorphization process, requires high-power ultrafast pulses that could be challenging to implement for wafer-scale heterostructures^32^. Alternatively, because of material compatibility, we can utilize standard nanofabrication processes to pattern large-scale heterostructures into an array of small-scale units with dimensions of  ~10 s of *μ*m × ~10 s of *μ*m to reduce the pulse power (Supplementary Fig. 26a). Each unit can be individually and electrically addressed by applying electrical signals to the bottom aligned CNT film using conventional electronics as demonstrated in prior and our recent works^19–21,44^. CD responses can be probed either in individual units with CD microscopy or collectively in the array with conventional CD spectrometers. We performed COMSOL simulations to analyze the temperature transient time response in a unit with lateral dimensions of 30 *μ*m and the same stack structural parameters in Fig. 3d when a short voltage pulse was applied (see Methods). The GST temperature reached above the melting temperature (>600 °C) at the pulse end (Supplementary Fig. 26b), and decayed fast with a fitting fast (slow) time constant *τ*_1_ ~ 290 ns (*τ*_2_ ~ 5.0 *μ*s); see Supplementary Fig. 26c. The fast decay is due to the large in-plane thermal conductivity along CNTs and the slow decay is due to the heat dissipation in the perpendicular direction^45^. Note that in practice the heterostructure needs to be in a vacuum chamber to avoid CNT oxidation at the GST melting temperature. After  ~3.2 *μ*s the temperature dropped below  ~150 °C, lower than the crystallization temperature to avoid re-crystallization. The simulation time response is quicker than that in our recent work^44^, where we experimentally demonstrated the bidirectional switching of a GST film of the same size as simulations. In addition, based on device dimensions and the electrical conductivity and thickness of aligned CNT films, we estimated the voltage height of the pulse as  ~14.8 V, which can be feasibly achieved using a simple electronic circuit (Supplementary Fig. 26d). Hence, this spatial-light-modulator-type array of heterostructure units (Supplementary Fig. 26a) offers a viable device architecture, enabling not only the bidirectional electrical tunability between crystalline and amorphous phases but also the spatial programming of chiroptical responses.

## Discussion

We have implemented a full stack of a versatile hardware platform of a programmable chiroptical heterostructure containing twisted aligned CNT and GST films and corresponding design software infrastructure based on ML frameworks including differentiable programming and derivative-free Bayesian optimization. The heterostructure is scalable not only in lateral dimensions to a wafer scale but also in the vertical thickness dimension to a large number of stacking layers. We have demonstrated the design and implementation of heterostructures to dynamically program both reciprocal and nonreciprocal CD responses, enabling the discovery of novel phenomena and the development of high-performance devices. The demonstrated 1D CNT and GST materials in the heterostructure can be expanded to a large library of 1D nanomaterials, PCMs, and electro-optic materials. Further, aligned CNT films are multifunctional and also serve as electrodes for switching PCMs. Although the required switching power is large due to the wafer-scale size of the heterostructure, the creation of microscopic planar structures on the heterostructure can substantially reduce switching power, allow reliable multilevel and bidirectional switching, improve cyclability, and enrich device functionalities. Non-contact optical approaches can induce PCM phase transitions without fabricating electrodes. For proof-of-concept, we employed a continuous-wave commercial laser writer to directly generate crystalline GST patterns on an amorphous GST film but the re-amorphization was not achievable (Methods and Supplementary Fig. 27). Instead, scanning ultrafast optical pulses can enable bidirectional phase transitions in small-size^18,46–48^ and wafer-scale GST devices^49^.

## Methods

### Shaking-assisted vacuum filtration (SAVF)

The CNT powder used was purchased from Carbon Solutions, Inc. (product number P2, with a purity  >90 wt%) and synthesized using the arc-discharge method. To prepare aqueous dispersions, 8 mg of CNT powder was mixed with 20 mL of an aqueous 0.5% w/w sodium deoxycholate solution, and then was sonicated using an ultrasonic tip horn sonicator (QSonica Q125) for 45 min at an output power of 21 W. Afterward, the sonicated suspension was purified by ultracentrifugation at 247, 104 × *g* for 2 h to remove large bundles. The supernatant was collected and diluted 27 times before undergoing vacuum filtration using a 1 inch filtration system (MilliporeSigma) with 100 nm-pore-size filter membranes (Whatman Nuclepore Track-Etched polycarbonate hydrophilic membranes, MilliporeSigma). The whole filtration system was placed on a linear shaker (Scilogex SCI-L180-Pro LCD digital linear shaker) as illustrated in Supplementary Fig. 1. Instead of a conventional cylindrical funnel, a funnel with a square-shaped cross-section was fabricated using a 3D printer (Elegoo Saturn 3D Printer). The lateral dimension of the square was 1 cm. Hence, the linear shaking direction was found to be the alignment direction of obtained aligned CNT films. The linear shaker shook the filtration system at 200 RPM during the first 15 min of the filtration process. Afterward, the standard filtration process continued without shaking. Close to the end of filtration, the vacuum pump was turned on to fix the alignment structure on the membrane. More details can be found in ref. ^50^. Supplementary Fig. 1 shows a photo and scanning electron microscopy image of the obtained film, confirming a good alignment along the shaking direction. The obtained film can be transferred onto the desired substrate for characterization and heterostructure fabrication using a wet transfer method. Specifically, a small droplet of water was first placed on the target substrate. The CNT film on the polycarbonate filter membrane was placed with the CNT side in contact with the wet substrate and the polycarbonate side on top. Once the water between the CNT film and the substrate evaporated, the top polycarbonate layer was removed by immersing the sample in a chloroform solution. The sample was finally cleaned with isopropanol.

### TMM calculations

A 4 × 4 transfer matrix method was developed to calculate chiroptical responses of the heterostructure containing anisotropic non-magnetic materials under normal incidence. The details can be found in ref. ^51^ and our prior work^25^. Briefly, as illustrated in Supplementary Fig. 2, a layer of an isotropic material was modeled with a scalar dielectric function *ε* and a thickness *t*. A layer of anisotropic material was modeled with a 2 × 2 dielectric function tensor ***ε***, a twist angle *θ*, and a thickness. Specifically, for an anisotropic material with orthogonal principal axes, such as the directions parallel and perpendicular to CNT alignment, there are four eigenmodes, which are *s*-wave forward, *s*-wave backward, *p*-wave forward, and *p*-wave backward modes, respectively. In the definition of the coordinate systems, waves propagate along the *z*-axis and *x* and *y* axes are in the heterostructure plane. $\hat{{{{\bf{x}}}}}$, $\hat{{{{\bf{y}}}}}$, and $\hat{{{{\bf{z}}}}}$ are defined as unit vectors along *x*-, *y*-, and *z*-axes, respectively. The transmitted field (*E*_*t*,*s*_, *E*_*t*,*p*_), reflected field (*E*_*r*,*s*_, *E*_*r*,*p*_), and the incident field (*E*_*i*,*s*_, *E*_*i*,*p*_) can be related through

1 $$\left(\begin{array}{c}{E}_{t,s}\\ 0\\ {E}_{t,p}\\ 0\end{array}\right)=Q\left(\begin{array}{c}{E}_{i,s}\\ {E}_{r,s}\\ {E}_{i,p}\\ {E}_{r,p}\end{array}\right)=\left(\begin{array}{cccc}{Q}_{11}&{Q}_{12}&{Q}_{13}&{Q}_{14}\\ {Q}_{21}&{Q}_{22}&{Q}_{23}&{Q}_{24}\\ {Q}_{31}&{Q}_{32}&{Q}_{33}&{Q}_{34}\\ {Q}_{41}&{Q}_{42}&{Q}_{43}&{Q}_{44}\\ \end{array}\right)\left(\begin{array}{c}{E}_{i,s}\\ {E}_{r,s}\\ {E}_{i,p}\\ {E}_{r,p}\end{array}\right),$$

where *Q* can be written as a product of a series of *D* and *P* matrices. Specifically, for a stack with *N* layers,

2 $$Q={D}_{N+1}^{-1}{D}_{N}{P}_{N}{D}_{N}^{-1}{D}_{N-1}{P}_{N-1}{D}_{N-1}^{-1}\cdot \cdot \cdot {D}_{1}{P}_{1}{D}_{1}^{-1}{D}_{0}$$

with

3 $${D}_{j}=\left(\begin{array}{cccc}\sin {\theta }_{j}&\sin {\theta }_{j}&\cos {\theta }_{j}&\cos {\theta }_{j}\\ -{n}_{{s}_{j}}\sin {\theta }_{j}&{n}_{{s}_{j}}\sin {\theta }_{j}&-{n}_{{p}_{j}}\cos {\theta }_{j}&{n}_{{p}_{j}}\cos {\theta }_{j}\\ {n}_{{s}_{j}}\cos {\theta }_{j}&-{n}_{{s}_{j}}\cos {\theta }_{j}&-{n}_{{p}_{j}}\sin {\theta }_{j}&{n}_{{p}_{j}}\sin {\theta }_{j}\\ \cos {\theta }_{j}&\cos {\theta }_{j}&-\sin {\theta }_{j}&-\sin {\theta }_{j}\\ \end{array}\right)$$

and

4 $${P}_{j}=\left(\begin{array}{cccc}{e}^{{{{\rm{i}}}}{k}_{j,1}{t}_{j}}&0&0&0\\ 0&{e}^{{{{\rm{i}}}}{k}_{j,2}{t}_{j}}&0&0\\ 0&0&{e}^{{{{\rm{i}}}}{k}_{j,3}{t}_{j}}&0\\ 0&0&0&{e}^{{{{\rm{i}}}}{k}_{j,4}{t}_{j}}\end{array}\right),$$

for *j*-th layer and *j* ∈ [0, *N* + 1]. Here, for the *j*-th layer, *t*_*j*_ is the thickness, ${k}_{j,1}=-{k}_{j,2}={k}_{0}{n}_{{s}_{j}}$, ${k}_{j,3}=-{k}_{j,4}={k}_{0}{n}_{{p}_{j}}$, ${n}_{{s}_{j}}$ is the refractive index along the *s*_*j*_ axis, ${n}_{{p}_{j}}$ is the refractive index along the *p*_*j*_ axis, *θ*_*j*_ is the angle of the *s*_*j*_ axis with respect to the *x*-axis in counterclockwise rotation, and *k*_0_ is vacuum wavevector. For layers with isotropic materials, such as the input and output layers, the principal axes are chosen to be ${s}_{0}={s}_{N+1}=\hat{{{{\bf{x}}}}}$ and ${p}_{0}={p}_{N+1}=\hat{{{{\bf{y}}}}}$. For layers with anisotropic materials, the dielectric function tensor ***ε***_*j*_ in *xy* coordinate systems can be written as

5 $${\varepsilon }_{j}={R}_{j}\left(\begin{array}{cc}{\varepsilon }_{{s}_{j}}&0\\ 0&{\varepsilon }_{{p}_{j}}\end{array}\right){{R}_{j}}^{-1},$$

6 $${R}_{j}=\left(\begin{array}{cc}\cos {\theta }_{j}&-\sin {\theta }_{j}\\ \sin {\theta }_{j}&\cos {\theta }_{j}\end{array}\right),$$

with ${n}_{{s}_{j}}=\sqrt{{\varepsilon }_{{s}_{j}}}$ and ${n}_{{p}_{j}}=\sqrt{{\varepsilon }_{{p}_{j}}}$. As a result, we can obtain transmission and reflection coefficients in terms of the matrix elements of *Q* as

7 $${r}_{ss}={\left.\frac{{E}_{r,s}}{{E}_{i,s}}\right| }_{{E}_{i,p}=0}=\frac{{Q}_{24}{Q}_{41}-{Q}_{21}{Q}_{44}}{{Q}_{22}{Q}_{44}-{Q}_{24}{Q}_{42}},$$

8 $${r}_{sp}={\left.\frac{{E}_{r,p}}{{E}_{i,s}}\right| }_{{E}_{i,p}=0}=\frac{{Q}_{21}{Q}_{42}-{Q}_{22}{Q}_{41}}{{Q}_{22}{Q}_{44}-{Q}_{24}{Q}_{42}},$$

9 $${t}_{ss}={\left.\frac{{E}_{t,s}}{{E}_{i,s}}\right| }_{{E}_{i,p}=0}={Q}_{11}+\frac{{Q}_{12}({Q}_{24}{Q}_{41}-{Q}_{21}{Q}_{44})+{Q}_{14}({Q}_{21}{Q}_{42}-{Q}_{22}{Q}_{41})}{{Q}_{22}{Q}_{44}-{Q}_{24}{Q}_{42}}$$

10 $${t}_{sp}={\left.\frac{{E}_{t,p}}{{E}_{i,s}}\right| }_{{E}_{i,p}=0}={Q}_{31}+\frac{{Q}_{32}({Q}_{24}{Q}_{41}-{Q}_{21}{Q}_{44})+{Q}_{34}({Q}_{21}{Q}_{42}-{Q}_{22}{Q}_{41})}{{Q}_{22}{Q}_{44}-{Q}_{24}{Q}_{42}}\,.$$

11 $${r}_{ps}={\left.\frac{{E}_{r,s}}{{E}_{i,p}}\right| }_{{E}_{i,s}=0}=\frac{{Q}_{24}{Q}_{43}-{Q}_{23}{Q}_{44}}{{Q}_{22}{Q}_{44}-{Q}_{24}{Q}_{42}},$$

12 $${r}_{pp}={\left.\frac{{E}_{r,p}}{{E}_{i,p}}\right| }_{{E}_{i,s}=0}=\frac{{Q}_{23}{Q}_{42}-{Q}_{22}{Q}_{43}}{{Q}_{22}{Q}_{44}-{Q}_{24}{Q}_{42}},$$

13 $${t}_{pp}={\left.\frac{{E}_{t,s}}{{E}_{i,p}}\right| }_{{E}_{i,s}=0}={Q}_{33}+\frac{{Q}_{32}({Q}_{24}{Q}_{43}-{Q}_{23}{Q}_{44})+{Q}_{34}({Q}_{23}{Q}_{42}-{Q}_{22}{Q}_{43})}{{Q}_{22}{Q}_{44}-{Q}_{24}{Q}_{42}},$$

14 $${t}_{ps}={\left.\frac{{E}_{t,p}}{{E}_{i,p}}\right| }_{{E}_{i,s}=0}={Q}_{13}+\frac{{Q}_{12}({Q}_{24}{Q}_{43}-{Q}_{23}{Q}_{44})+{Q}_{14}({Q}_{23}{Q}_{42}-{Q}_{22}{Q}_{43})}{{Q}_{22}{Q}_{44}-{Q}_{24}{Q}_{42}}.$$

The input light of any other polarization states can be represented as a linear combination of *E*_*i*,*s*_ and *E*_*i*,*p*_. For example, LCP light can be represented as 0.5*E*_*i*,*s*_ + 0.5i*E*_*i*,*p*_, RCP light can be represented as 0.5*E*_*i*,*s*_–0.5i*E*_*i*,*p*_. Any linearly polarized with the angle between polarization direction and *s*-wave polarization direction, *α*, can be represented as cos(*α*)*E*_*i*,*s*_ + sin(*α*)*E*_*i*,*p*_. Hence, the output fields and their amplitude and phase can be calculated using the input field vector in Eq. (1). The transfer matrix method can be further expanded to simulate and design 2D periodic structures following the formalism of rigorous coupled-wave analysis^52^. Since the operations described above are all matrix-matrix multiplications, PyTorch (version 1.9.0) was used to implement the TMM calculation framework with a built-in backpropagation algorithm for gradient descent-based optimization. An Nvidia GeForce RTX 3090 GPU card with CUDA version 11.1 was used to accelerate calculations.

### Four-configuration CD simulation and measurement

Theoretical analyses^8,9^ have shown that the observed CD signals from solid-state samples (CD_obs_) can be described as CD_obs_ = CD_iso_ + 0.5(${{{{\rm{LD}}}}}^{{\prime} }$LB  − LD${{{{\rm{LB}}}}}^{{\prime} }$) + *α*_c_(${{{{\rm{LD}}}}}^{{\prime} }$ sin(2*θ*) - LDcos(2*θ*)). The first term CD_iso_ is the intrinsic component of CD, which is from excitonic transitions in CNTs and is isotropic and independent of sample orientation. The second LDLB term is from the interference of the sample’s LD and LB responses. This term is independent of instrumental faults and not an artifact. It represents a real, perfectly reproducible differential attenuation of LCP and RCP light. This LDLB term is invariant upon sample rotation around the axis perpendicular to the sample plane but inverts the sign under sample flipping. The third term artifact comes from the coupling of residual birefringence in the instrument and LD and LB of samples. This artifact term inverts the sign under 90° sample rotation around the axis perpendicular to the sample plane. Based on these properties, a four-configuration protocol was employed to extract the CD_iso_ and LDLB terms. Specifically, when the sample was positioned as illustrated in Supplementary Fig. 3, four CD spectra were calculated or measured and denoted as CD_m1_, CD_m2_, CD_m3_, and CD_m4_. The average spectra under in-plane rotation, which are 0.5(CD_m1_ + CD_m2_) or 0.5(CD_m3_ + CD_m4_), can remove the third term. Hence, the term 0.5(CD_m1_ + CD_m2_) (i.e., the observed CD, CD_obs_, from the front side) corresponds to CD_iso_ + 0.5(${{{{\rm{LD}}}}}^{{\prime} }$LB  − LD${{{{\rm{LB}}}}}^{{\prime} }$) and the term 0.5(CD_m3_ + CD_m4_) (i.e., the observed CD, CD_obs_, from the back side) corresponds to CD_iso_ − 0.5(${{{{\rm{LD}}}}}^{{\prime} }$LB  − LD${{{{\rm{LB}}}}}^{{\prime} }$). Hence, CD_iso_ = 0.25(CD_m1_ + D_m2_ + CD_m3_ + CD_m4_) and 0.5(${{{{\rm{LD}}}}}^{{\prime} }$LB  − LD${{{{\rm{LB}}}}}^{{\prime} }$) = 0.25(CD_m1_ + CD_m2_ − CD_m3_ − CD_m4_).

### Linear- and circular-polarization dependent optical spectroscopy

CD spectra and average absorption spectra of LCP and RCP light were measured using a Jasco J-810 CD spectrometer, covering a wavelength range of 200−900 nm. Linearly polarized absorption spectra were measured using an ultraviolet-visible-near-infrared (UV-vis-NIR) spectrometer (Perkin Elmer Lambda 950 UV-vis-NIR) equipped with an automatically controlled rotating broadband polarizer in the same wavelength range. The incident beam with a diameter of 2 mm was defined by a customized 3D-printed sample holder, and was the same in all measurements. In the design and fitting of heterostructures, the thicknesses of aligned CNT films were benchmarked by comparing the peak average absorption of LCP and RCP light in the UV range with that of a reference sample whose thickness was measured using an atomic force microscope (Parksystems NX20)^25^. The peak average UV absorption was assumed to be proportional to the sample thickness.

### Measurement of dielectric functions of aligned CNTs

The dielectric functions parallel and perpendicular to the CNT alignment direction from the UV to NIR ranges were modeled as a summation of Voigt functions^53^

15 $${\varepsilon }_{s,p}(\omega )={\varepsilon }_{\infty,s,p}+{\sum }_{n=1}^{N}{\left.{C}_{{{{\rm{V}}}},s,p}(\omega )\right\vert }_{{A}_{n},{\omega }_{0,n},{\gamma }_{{{{\rm{L}}}},n},{\gamma }_{{{{\rm{G}}}},n}}$$

with

16 $${\left.{C}_{{{{\rm{V}}}}}(\omega )\right\vert }_{A,{\omega }_{0},{\gamma }_{{{{\rm{L}}}}},{\gamma }_{{{{\rm{G}}}}}}=-A\frac{{{{\rm{Im}}}}(F(x-{x}_{0}-{{{\rm{i}}}}y)+F(x+{x}_{0}+{{{\rm{i}}}}y))}{{{{\rm{Re}}}}(F({{{\rm{i}}}}y))}$$

17 $$+{{{\rm{i}}}}A\frac{{{{\rm{Re}}}}(F(x-{x}_{0}+{{{\rm{i}}}}y)-F(x+{x}_{0}+{{{\rm{i}}}}y))}{{{{\rm{Re}}}}(F({{{\rm{i}}}}y))}$$

and

18 $$x=\frac{2\sqrt{{{{\rm{\ln }}}}2}}{{\gamma }_{{{{\rm{G}}}}}}\omega,{x}_{0}=\frac{2\sqrt{{{{\rm{\ln }}}}2}}{{\gamma }_{{{{\rm{G}}}}}}{\omega }_{0},y=\frac{{\gamma }_{{{{\rm{L}}}}}\sqrt{{{{\rm{\ln }}}}2}}{{\gamma }_{{{{\rm{G}}}}}},$$

where *ω* is angular frequency, *A* is amplitude factor, *ω*_0_ is resonance frequency, *γ*_L_ is Lorentz linewidth, *γ*_G_ is Gaussian linewidth, and *F* is Faddeeva function. *N* was selected as 5. Hence, for each polarization (*s* or *p*), there were 21 fitting parameters, including {*A*_*n*_, *ω*_0,*n*_, *γ*_L,*n*_, *γ*_G,*n*_, *n* = 1 − 5} and *ϵ*_*∞*_, and 42 fitting parameters in total for both polarizations. These fitting parameters were uniquely determined by simultaneously fitting six experimentally measured spectra, including linear-polarization absorption spectra for one-layer aligned CNT film, and averaged absorption of LCP and RCP light, and CD spectra of twisted two-layer and three-layer stacks with a twist angle of 30°, as shown in Supplementary Fig.4. The fitting wavelength range was from 600 nm to 800 nm.

### Measurement of dielectric functions of GST films

The film thickness and optical constants (refractive index *n* and extinction coefficient *k*) of GST films were measured using a J.A. Woollam RC2 Spectroscopic Ellipsometer over a wavelength range of 190–1700 nm. The measurements were conducted over a wide spectral range from UV to NIR and were analyzed using CompleteEASE software. In this procedure, polarized light was reflected off the sample surface, and the change in polarization was measured as two quantities: Ψ and *Δ*. Ψ represents the amplitude ratio and *Δ* represents the phase difference. A model describing the sample’s physical structure (layers and materials) was created and adjusted to fit the measured Ψ and *Δ* values through regression analysis. The software calculated results based on the model, and the model parameters (e.g., layer thicknesses, optical constants) were varied to minimize the mean-squared error between experimental and calculation results. The fitting process yielded the film thickness and the optical constants of the material. These optical constants include the real and imaginary parts of refractive indices, or alternatively, the real and imaginary parts of the dielectric function *ε*.

### Backpropagation and Bayesian optimization algorithms

The Adam optimizer in PyTorch was employed for the backpropagation algorithm. The learning rate was adjusted in a range of 0.01 to 0.2. For Bayesian optimization, the results shown in Fig. 3c were obtained without the bound of the twist angle between aligned CNT films. For the results shown in Supplementary Fig. 6, the twist angle was bounded in a range of 22.5–67.5°. The accumulated average over all epochs was plotted. For simultaneous optimizations of *Δ*CD_iso_ and *Δ*LDLB using the backpropagation algorithm, the optimization target was defined to maximize a linear combination of *Δ*CD_iso_ and *Δ*LDLB, expressed as *βΔ*CD_iso_ + (1 − *β*)*Δ*LDLB, where *β* was a parameter in a range of 0 to 1. Hence, the loss function in the backpropagation algorithm was defined as the most negative absolute value of this expression in the wavelength range of 600–800 nm. The value of *β* was swept in the range and the backpropagation was performed for each *β*. For each *β*, *Δ*CD_iso_ and *Δ*LDLB spectra were calculated from the heterostructure with the structural parameters obtained at each epoch. Supplementary Fig. 7a, b display 2D plots of simultaneously optimized *Δ*CD_iso_ and *Δ*LDLB as a function of *β* and epochs, and Supplementary Fig. 7c shows obtained *Δ*CD_iso_ and *Δ*LDLB as functions of *β* at the end of optimization. *Δ*CD_iso_ increases and reaches the maximum value at *β* = 1, corresponding to the case of optimizing *Δ*CD_iso_ only. In contrast, *Δ*LDLB decreases and has the maximum value at *β* = 0, corresponding to the case of optimizing *Δ*LDLB only.

### Heterostructure fabrication

The heterostructure fabrication process consists of (i) transfer of aligned CNT films, (ii) deposition of GST and other dielectrics, and (iii) twist-stacking of aligned CNT films. The substrate was fused silica for broadband transparency. For (i), aligned CNT films were produced through the SAVF method as described in the first method section. The alignment direction was determined by the linear shaking direction. The produced film was typically cut into multiple pieces for twist-stacking and transferred onto the substrate or heterostructure using the wet transfer process as described in the first method section. For (ii), SiO_2_ films were deposited using a Denton Discovery 18 Sputtering System at an argon pressure of 6 mTorr with a power setting of 100 W. GST films were deposited using the same system at an argon pressure of 4.5 mTorr and a power setting of 35 W. For (iii), to facilitate twist-stacking, the heterostructure was put onto a transparent protractor, which was back-illuminated by a light-emitting-diode panel. An aligned CNT film was placed on top of the heterostructure and the orientation was rotated to a specific angle relative to transferred aligned CNT films before. The film was then transferred using the same wet transfer process. To induce the phase transition in GST films, the heterostructure was placed on a hotplate (Corning hotplate and stirrer with digital display) at set temperatures and left for a few minutes for a complete phase transition.

### Spectral analysis with artificial GST films

To understand the contributions of the change in GST’s real and imaginary parts of optical constants to the tunability of CD_iso_ and LDLB, simulations and optimizations were performed by artificially assuming the optical properties of GST films and comparing their results with the actual GST film. Two cases were considered: Case 1 represents the change in complex-valued dielectric function (*ε*_PCM_) as shown in Fig. 2c and Case 2 represents the change in complex-valued refractive index as shown in Supplementary Fig. 5. In Case 1, for real-part-only tuning, the imaginary part of the dielectric function under the crystalline phase (Imag(*ε*_PCM,c_)) was kept the same as the amorphous phase (Imag(*ε*_PCM,a_)) while the real parts were tunable from Real(*ε*_PCM,a_) to Real(*ε*_PCM,c_). Similarly, for imaginary-part-only tuning, Real(*ε*_PCM,c_) was kept the same as Real(*ε*_PCM,a_) while Imag(*ε*_PCM,a_) was changed to Imag(*ε*_PCM,c_) when the amorphous phase transitioned to the crystalline phase. In Case 2, the same operations were performed on the complex-valued refractive index to construct artificial optical properties of GST films. For real-part-only tuning, the imaginary part of the refractive index under the crystalline phase (*κ*_*c*_) was kept the same as the amorphous phase (*κ*_*a*_) while the real parts were tunable from *n*_*a*_ to *n*_*c*_. For imaginary-part-only tuning, *n*_*c*_ was kept the same as *n*_*a*_ while *κ*_*a*_ was changed to *κ*_*c*_ under the phase transition. For Case 2, Supplementary Fig. 14a, b display the corresponding complex-valued dielectric functions with *n* and *κ* tuning, respectively, by taking the square of complex-valued refractive indices.

For Case 1, Supplementary Fig. 15a, b show the simulation CD_iso_ and absorption spectra of the heterostructure with the optimized structural parameters in Fig. 3d for the actual GST film, Supplementary Fig. 15c, d show the corresponding simulation spectra for the real-part-only tuning artificial GST film, and Supplementary Fig. 15e, f show the spectra for the imaginary-part-only tuning artificial GST film. By comparison, the major contribution to the tunability of CD_iso_ comes from the tunable imaginary part of the dielectric function. Similarly, Supplementary Fig. 16a–f summarizes the LDLB and absorption spectra of the heterostructure with the structural parameters in Fig. 3e for the actual, real-part-only tuning, and imaginary-part-only tuning GST films. The major contribution to the tunability of LDLB also originates from the tunable imaginary part of the dielectric function. For Case 2, Supplementary Fig. 17a–f and Supplementary Fig. 18a–f summarize CD_iso_, LDLB, and corresponding absorption spectra with the actual GST film and artificial GST films with real-part-only tuning or imaginary-part-only tuning of refractive indices. Also, the major contribution to the tunability of CD_iso_ and LDLB is from the tunable imaginary parts.

In addition, gradient-based backpropagation optimizations were performed to design heterostructures with artificial GST films with real-part-only or imaginary-part-only tuning in Case 1 and Case 2. Supplementary Fig. 19 summarizes training curves for optimizing *Δ*CD_iso_ and *Δ*LDLB under conditions of different artificial GST films. It turns out that the tunability of imaginary parts of either the dielectric function or the refractive index leads to a larger tunable range that is closer to the optimized values with the actual GST film. Hence, for the actual GST film with simultaneous real and imaginary parts tuning, the optimization process tends to favor structures similar to those obtained with imaginary-part-only-tuning artificial GST films. Further, Supplementary Figs. 20, 21 summarize the CD_iso_, LDLB, and absorption spectra of the optimized structures with real-part-only-tuning and imaginary-part-only-tuning artificial GST films in Case 1 and Case 2, respectively. The spectra shapes were also similar to those obtained with the actual GST film shown in Supplementary Fig. 15a, b, 16a, b, 17a, b, 18a, b, further highlighting the tunable imaginary parts of the dielectric function and refractive index are dominant contributors.

### Layer scalability analysis with artificial low-loss GST films

To mimic low-loss GST films, as mentioned before, in Case 1, Imag(*ε*_PCM,c_) and Imag(*ε*_PCM,a_) of the GST film were artificially set as zero in the wavelength range of 600−800 nm. In Case 2, *κ*_*a*_ and *κ*_*c*_ were set as zero in the same wavelength range. The backpropagation algorithm was used to design heterostructures with various numbers of aligned CNTs for optimizing *Δ*CD_iso_ and *Δ*LDLB. Supplementary Fig. 25 summarizes the maximum *Δ*CD_iso_ and *Δ*LDLB as a function of the CNT layer number and corresponding maximum attenuation in the wavelength range that CD_iso_ and LDLB display tunability. In Case 1, as shown in Supplementary Fig. 25a–d, the upper scaling limit was 9 before reaching the benchmark attenuation limit of 5. In contrast, with actual GST optical properties, the upper scaling limit was 4 before reaching the benchmark. Further, the maximum *Δ*CD_iso_ and *Δ*LDLB were  ~24.3 deg and  ~20.7 deg because of lower loss and more layers. Similarly, in Case 2, as shown in Supplementary Fig. 25e, f, the upper scaling limit was 8 before reaching the benchmark, and the maximum *Δ*CD_iso_ and *Δ*LDLB were  ~20.1 deg and  ~16.3 deg.

### Finite element simulations

A 2D finite element simulation using COMSOL Multiphysics was conducted to analyze the temperature distribution of the heterostructure under applied voltage and current. Because of the limited computer memory resource and wafer-scale sample sizes, the finite element model and thermal excitation were simultaneously scaled down. Specifically, the thickness or vertical dimension of the heterostructure was kept unchanged, while the lateral dimension was scaled down to 500 × 500 nm^2^. The thicknesses of the layers from top to bottom were 20 nm for the top CNT film, 71 nm for SiO_2_, 11 nm for GST, 68 nm for SiO_2_, and 20 nm for the bottom CNT film. The thermal conductivity of CNTs along the tube axis was set as 43 W m^−1^ K^−1^ ^54^, and the GST thermal conductivity was set as 0.27 W m^−1^ K^−1^ ^55^. The heat source was a square wave with a duration of 5 *μ*s and a power of 3 × 10^−5^ W, resulting in a total heat energy of 1.5 × 10^−10^ J. This was scaled down from the injected heat energy into our fabricated device with a lateral size of of 5 mm × 5 mm, which was estimated with a power 1.1 W and phase transition time  ~1–2 ms.

The COMSOL simulations were also conducted for a small-scale device with lateral dimensions of 30 *μ*m and the same heterostructure structural parameters as those in Fig. 3d; see the illustration in Supplementary Fig. 26a. A short 500 ns long and 1.1 W square voltage pulse was applied to the bottom aligned CNT film. Supplementary Fig. 26b displays the spatial profile of the temperature at the end of the pulse. The simulation time-dependent temperature at the center of the GST film is shown in Supplementary Fig. 26c (red dots). The achieved peak temperature was  ~620 °C. A double exponential decay function, $a{e}^{-t/{\tau }_{1}}+b{e}^{-t/{\tau }_{2}}$, was used to fit the decay dynamics starting at the end of the applied pulse with *a*, *b*, *τ*_1_, and *τ*_2_ as fitting parameters (black dashed line). The obtained fast time constant *τ*_1_ was  ~290 ns and the slow time constant *τ*_2_ was  ~5.0 *μ*s. After  ~3.2 *μ*s the temperature dropped below  ~150 °C. Supplementary Fig.  26d shows an example electronic circuit to generate voltage pulses. An electrical pulse generated by a function generator was used to drive a transistor connected to a direct-current voltage source with an amplitude *V*_pp_ and a pulse with *V*_pp_ height was applied to the heterostructure device. If the electrical conductivity along CNT alignment direction was set as 2500 S/cm^50^, the CNT heater lateral dimension was set as 30 *μ*m, and the CNT heater thickness was set as 20 nm, *V*_pp_ was estimated as  ~14.8 V for a 500 ns long and 1.1 W pulse.

### Direct laser writing

A commercial laser writer (Heidelberg *μ* PG 101 Laser Pattern Generator) with a 405 nm-wavelength continuous-wave tunable-power laser was utilized to directly write crystalline GST patterns on an amorphous GST film by inducing the phase transition from amorphous to crystalline phases. The minimum writing feature was 1 *μ*m and the scan speed was 3 mm^2^ per minute. Supplementary Fig. 27a displays multiple writing rectangular patterns with dimensions of 10 *μ*m × 100 *μ*m, 20 *μ*m × 100 *μ*m, and 40 *μ*m × 100 *μ*m. Under low laser powers of 2 mW and 4 mW, the phase transition was induced. While under high laser powers of 6 mW, 8 mW, and 10 mW, the GST film was partially or completely removed. Further, for a crystalline GST film, only the film removal was observed and there was no phase transition from crystalline to amorphous phases, as shown in Supplementary Fig. 27b. Hence, it appears that slow laser heating can induce GST crystallization while ultrafast laser pulses are needed for GST re-amorphization.

## Supplementary information

Supplementary Information

Description of Additional Supplementary Information

Supplementary Movie 1

Transparent Peer Review file

## Source data

Source Data

## Data Availability

The data generated in this study are provided in the Source Data file. Source data are provided with this paper.
