# Supplementary material for: A Programmable Wafer-scale Chiroptical Heterostructure of Twisted Aligned Carbon Nanotubes and Phase Change Materials
Source: Nat Commun. 2025 May 14;16:4478. doi: 10.1038/s41467-025-59600-w (PMC12078695; doi:10.1038/s41467-025-59600-w)
Supplement: Supplementary file 1 — Supplementary Information [file 41467_2025_59600_MOESM1_ESM.pdf]

Supplementary Information: A Programmable Wafer-scale Chiroptical Heterostructure of Twisted Aligned Carbon Nanotubes and Phase Change Materials

Jichao Fan[1], Ruiyang Chen[1], Minhan Lou[1], Haoyu Xie[1], Nina Hong[2], Benjamin Hillam[1], Jacques Doumani[1,3], Yingheng Tang[1,4], Weilu Gao[1*]

[1]Department of Electrical and Computer Engineering, The University of Utah, Salt Lake City, UT, USA.
[2]J.A. Woollam Co., Inc., Lincoln, NE, USA.
[3]Department of Electrical and Computer Engineering, Rice University, Houston, TX, USA.
[4]Elmore Family School of Electrical and Computer Engineering, Purdue University, West Lafayette, IN, USA.

*Corresponding author(s). E-mail(s): weilu.gao@utah.edu;

Supplementary Table 1 Summary of optimized structural parameters. Source data are provided as a Source Data file.

Figure No.	Chiroptical responses	Structural parameters
Fig. 3d	ΔCD_{iso}	Thickness (nm): {CNT, SiO_2, GST, SiO_2, CNT} = {20, 71, 10.9, 67.7, 20} Twisted angle between CNTs: {45.6°}
Fig. 3e	ΔLDLB	Thickness (nm): {CNT, SiO_2, GST, SiO_2, CNT} = {20, 119.1, 12.5, 29.1, 20} Twisted angle between CNTs: {45.4°}
Fig. 4b	ΔCD_{iso}	Thickness (nm): {CNT, SiO_2, GST, SiO_2, CNT, SiO_2, GST, SiO_2, CNT} = {20, 121.5, 21.6, 115, 20, 112.9, 21, 130.8, 20} Twisted angle between CNTs: {44.2°, 88.7°}
Fig. 4c	ΔCD_{iso} (4L)	Thickness (nm): {CNT, SiO_2, GST, SiO_2, CNT, SiO_2, GST, SiO_2, CNT, SiO_2, GST, SiO_2, CNT} = {20, 117.4, 21.6, 108.2, 20, 111.9, 23.6, 112.5, 20, 108.1, 21.2, 123.1, 20} Twisted angle between CNTs: {41.7°, 81.8°, 110.6°}
Fig. 4c	ΔCD_{iso} (5L)	Thickness (nm): {CNT, SiO_2, GST, SiO_2, CNT, SiO_2, GST, SiO_2, CNT, SiO_2, GST, SiO_2, CNT, SiO_2, GST, SiO_2, CNT} = {20, 112.5, 21.9, 109.5, 20, 110.9, 22.1, 108.9, 20, 109.2, 22.3, 111.3, 20, 109.2, 21.5, 117.5, 20} Twisted angle between CNTs: {40.4°, 74.3°, 102.7°, 125.1°}
Fig. 4c	ΔCD_{iso} (6L)	Thickness (nm): {CNT, SiO_2, GST, SiO_2, CNT, SiO_2, GST, SiO_2, CNT, SiO_2, GST, SiO_2, CNT, SiO_2, GST, SiO_2, CNT, SiO_2, GST, SiO_2, CNT} = {20, 108.7, 22.6, 109.9, 20, 110, 21.8, 110.5, 20, 109.8, 20.9, 109.5, 20, 110.6, 22.1, 110.4, 20, 109.4, 22.3, 113.4, 20} Twisted angle between CNTs: {38.6°, 58.1°, 94.5°, 117.7°, 135.8°}
Fig. 4d	ΔLDLB (4L)	Thickness (nm): {CNT, SiO_2, GST, SiO_2, CNT, SiO_2, GST, SiO_2, CNT, SiO_2, GST, SiO_2, CNT} = {20, 102.5, 17.9, 67.4, 20, 108.6, 22.3, 110.6, 20, 119.8, 24.1, 120.4, 20} Twisted angle between CNTs: {120.7°, 59.2°, 89°}
Fig. 4d	ΔLDLB (5L)	Thickness (nm): {CNT, SiO_2, GST, SiO_2, CNT, SiO_2, GST, SiO_2, CNT, SiO_2, GST, SiO_2, CNT, SiO_2, GST, SiO_2, CNT} = {20, 132.9, 20.5, 112.5, 20, 118.3, 24.9, 117.3, 20, 99.2, 21.6, 88, 20, 84.8, 17.9, 41.7, 20} Twisted angle between CNTs: {29.4°, 86.9°, 71.6°, 39.4°}
Fig. 4d	ΔLDLB (6L)	Thickness (nm): {CNT, SiO_2, GST, SiO_2, CNT, SiO_2, GST, SiO_2, CNT, SiO_2, GST, SiO_2, CNT, SiO_2, GST, SiO_2, CNT, SiO_2, GST, SiO_2, CNT} = {20, 96, 21.7, 84.1, 20, 103.7, 22.6, 122.3, 20, 114.5, 21.4, 92, 20, 83.8, 21.3, 107.1, 20, 118.8, 24.7, 112.3, 20} Twisted angle between CNTs: {139.7°, 40.6°, 106.4°, 72.9°, 86.9°}

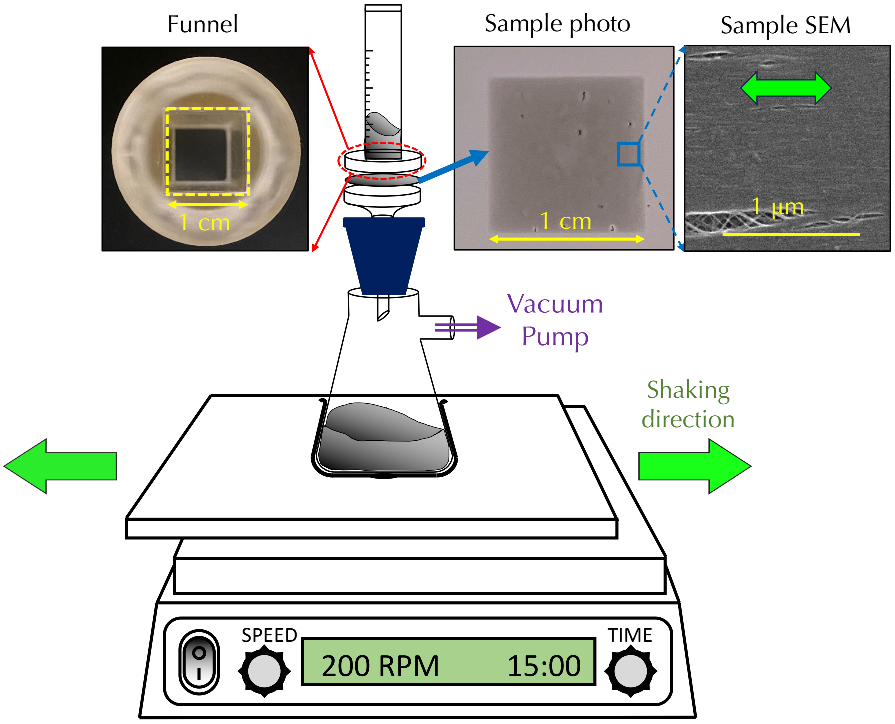

Supplementary Fig. 1 Illustration of the SAVF process, photo of a 3D-printed funnel with a square-shaped opening, photo of a fabricated square-shaped aligned CNT film, and a scanning electron microscopy (SEM) image showing alignment direction.

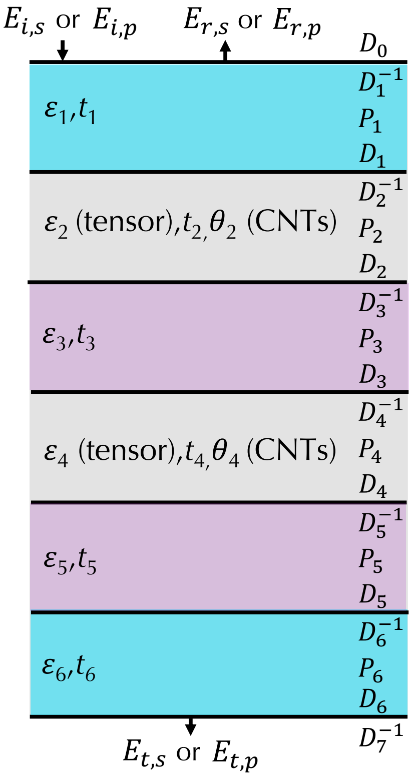

Supplementary Fig. 2 Illustration of transfer matrix method to calculate transmission and reflection coefficients of the chiroptical heterostructure containing anisotropic and isotropic materials.

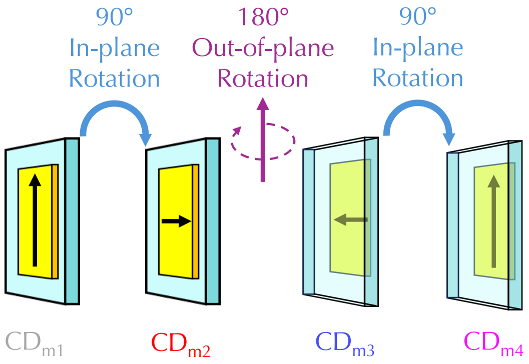

Supplementary Fig. 3 Illustration of simulating and experimentally measuring CD spectra under four sample configurations.

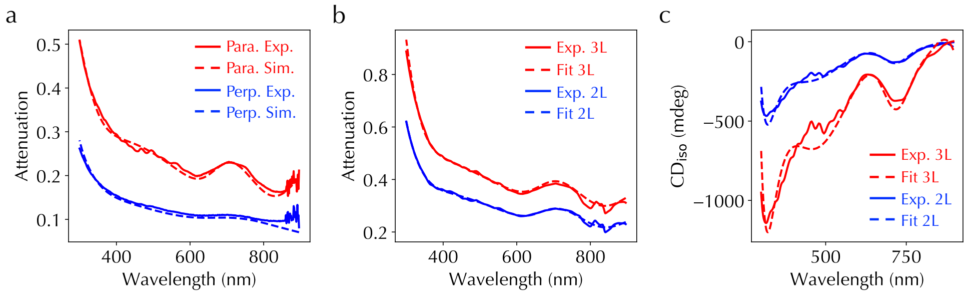

Supplementary Fig. 4 Measured (solid lines) and simulation (dashed lines) (a) linear-polarization-dependent absorption spectra under parallel polarization (red lines) and linear polarization (blue lines) for an aligned CNT film, (b) average absorption spectra of LCP and RCP light for 2-layer (blue lines) and 3-layer (red lines) twisted stacks containing only aligned CNT films, (c) CD_{iso} spectra of 2-layer (blue lines) and 3-layer (red lines) twisted stacks containing only aligned CNT films. Source data are provided as a Source Data file.

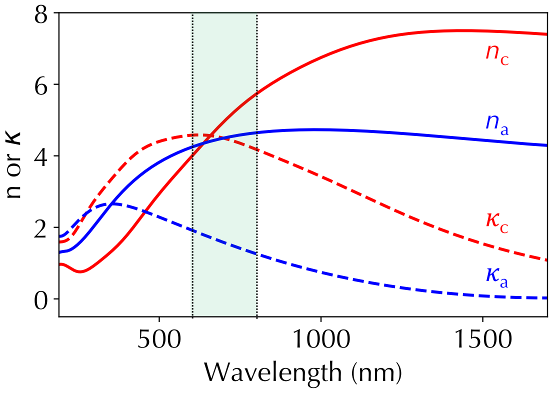

Supplementary Fig. 5 Complex-valued refractive index determined by spectroscopic ellipsometry. The red (blue) solid line is the real part of the refractive index, denoted as n, under the GST crystalline (amorphous) phase. The dashed red and blue lines are for the imaginary part, denoted as κ, under two phases. The shaded area indicates the wavelength range from 600 nm to 800 nm. Source data are provided as a Source Data file.

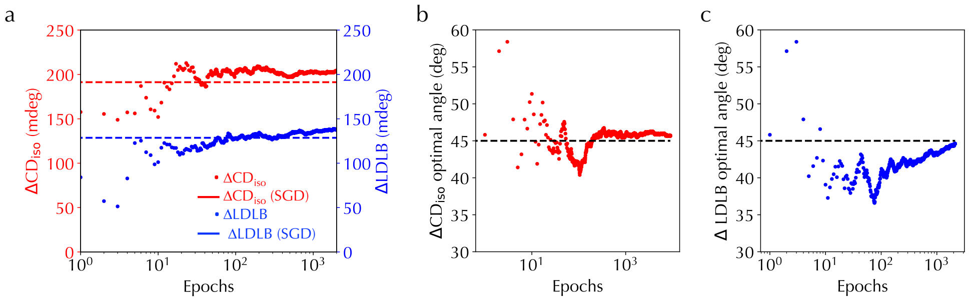

Supplementary Fig. 6 (a) Training curves of ΔCD_{iso} (red dots) and ΔLDLB (blue dots) using the derivative-free Bayesian optimization with a narrower range of twist angle between aligned CNTs, which is from 22.5° to 67.5° Red (blue) dashed lines indicate optimized values for ΔCD_{iso} and ΔLDLB in Fig. 3b of the main text. Obtained optimal twist angles using (b) differentiable programming (red dots) and (c) derivative-free Bayesian optimization (blue dots), respectively. The black lines in (b) and (c) indicate 45°. Source data are provided as a Source Data file.

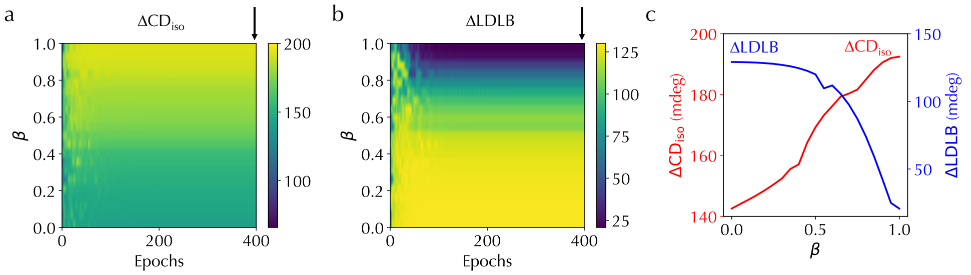

Supplementary Fig. 7 2D plots of simultaneously optimized (a) $\Delta\mathrm{CD}_{\mathrm{iso}}$ and (b) ΔLDLB as a function of a real-valued hyperparameter β and epochs. (c) $\Delta\mathrm{CD}_{\mathrm{iso}}$ (red line) and ΔLDLB (blue line) at the end of optimization as a function of β. Source data are provided as a Source Data file.

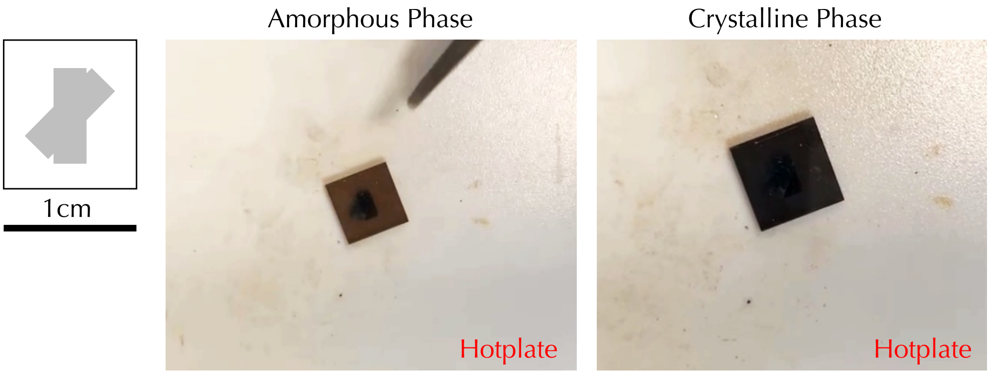

Supplementary Fig. 8 Illustration of the heterostructure sample size and photos showing the phase change on a hotplate. See Supplementary Video 1 for a full video.

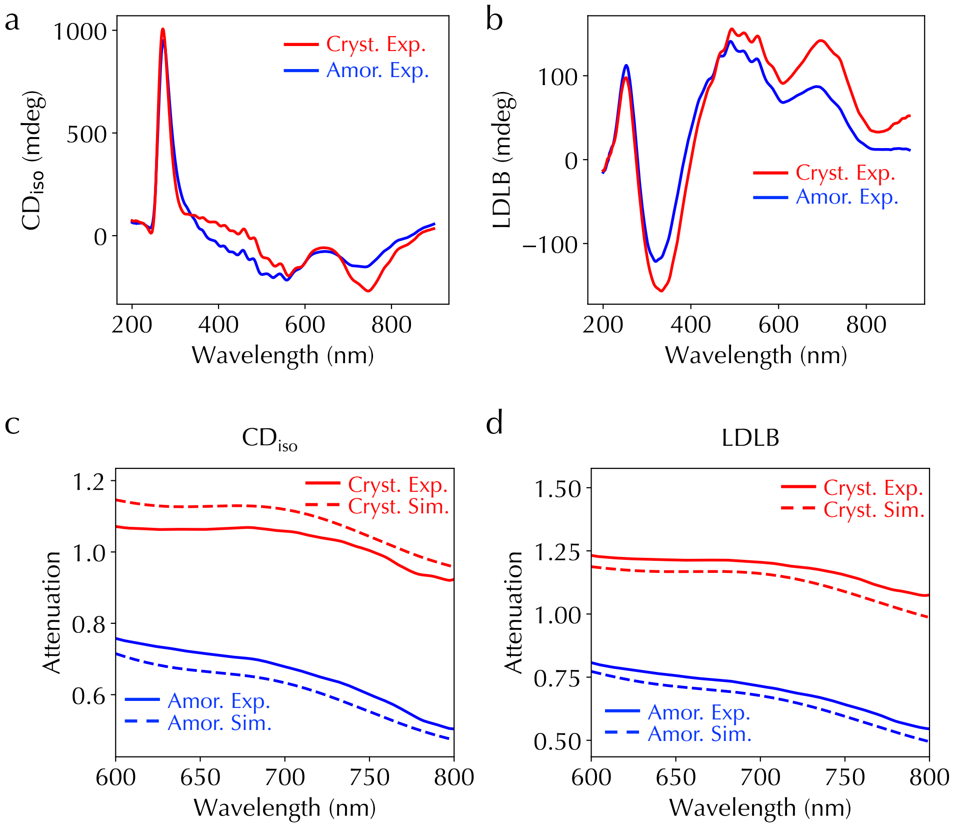

Supplementary Fig. 9 Measured broadband spectra of (a) CD_{iso}, (b) LDLB, and (c), (d) corresponding measured (solid lines) and simulation (dashed lines) average absorption spectra of LCP and RCP light under GST crystalline (red lines) and amorphous (blue lines) phases for the heterostructures in Fig. 3 of the main text. Source data are provided as a Source Data file.

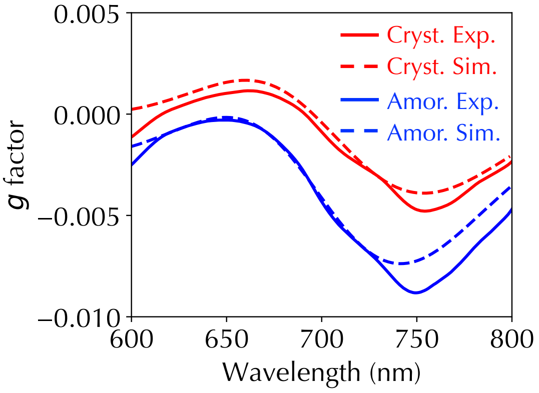

Supplementary Fig. 10 Measured (solid lines) and simulation (dashed lines) spectra of g factor under GST crystalline (red lines) and amorphous (blue lines) phases. Source data are provided as a Source Data file.

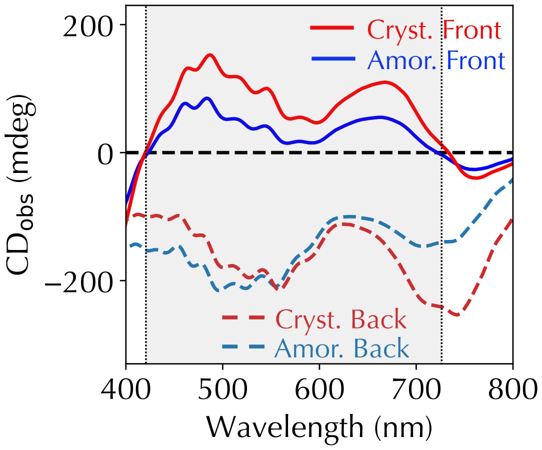

Supplementary Fig. 11 Observed CD (CD_{obs}) broadband spectra measured from the front (solid lines) and back (dashed lines) sides of the same heterostructure in Fig. 3 of the main text under GST crystalline (red lines) and amorphous (blue lines) phases. The gray shaded area indicates the polarity reversal range. Source data are provided as a Source Data file.

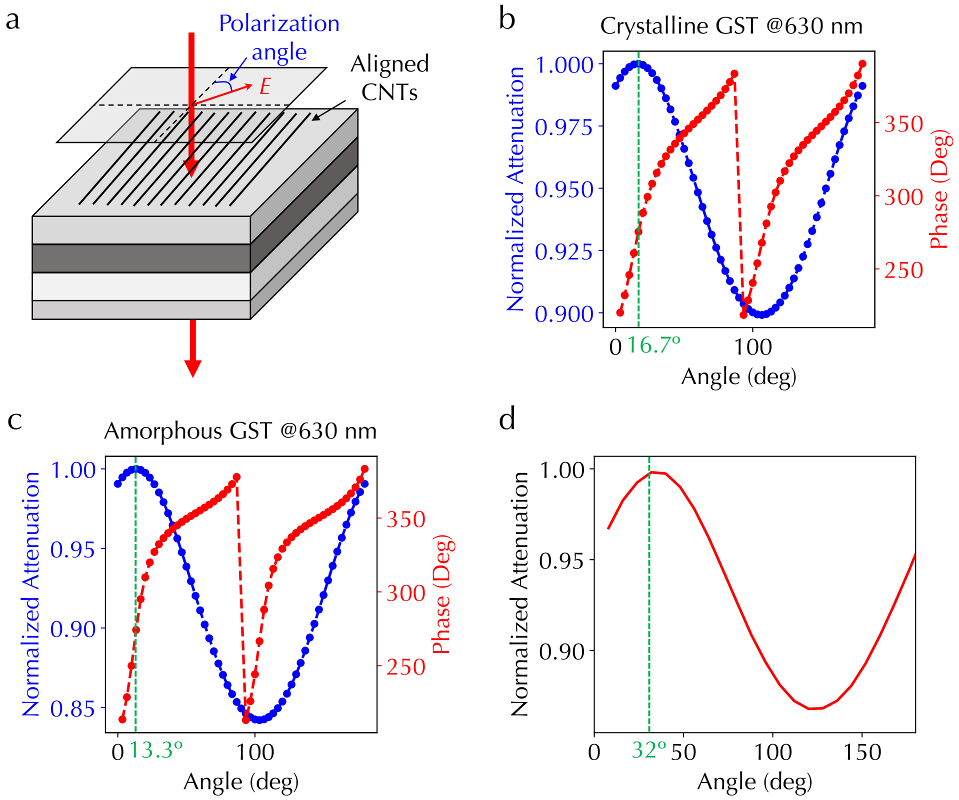

Supplementary Fig. 12 (a) Schematic of calculating linear-polarization-dependent transmitted intensity and the phase delay between transmitted light and input light in the heterostructure. The zero degree means that the orientation of first-layer aligned CNTs and the polarization direction are the same. Calculated normalized attenuation (blue dashed lines with blue dot markers) and phase delay (red dashed lines with red dot markers) as a function of polarization angle at 630 nm wavelength for GST (b) crystalline and (c) amorphous phases. Green lines indicate maximum attenuation positions. (d) Experimentally measured attenuation as a function of polarization angle. The green line indicates the maximum attenuation position. Source data are provided as a Source Data file.

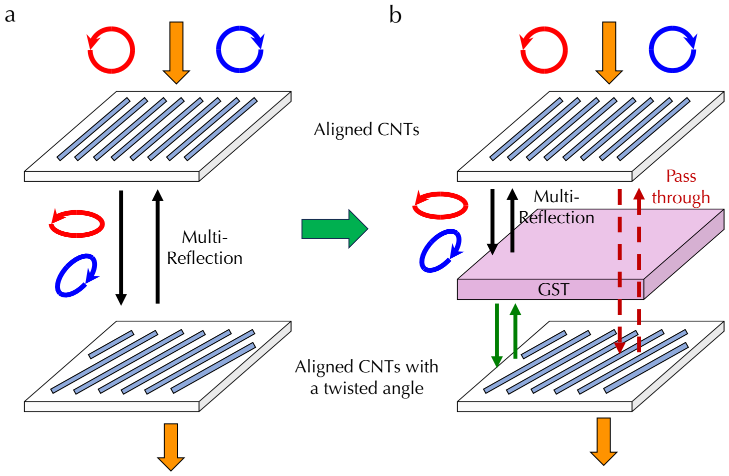

Supplementary Fig. 13 Illustrations of light polarization states and propagation in chiroptical heterostructures (a) without GST and (b) with a GST film.

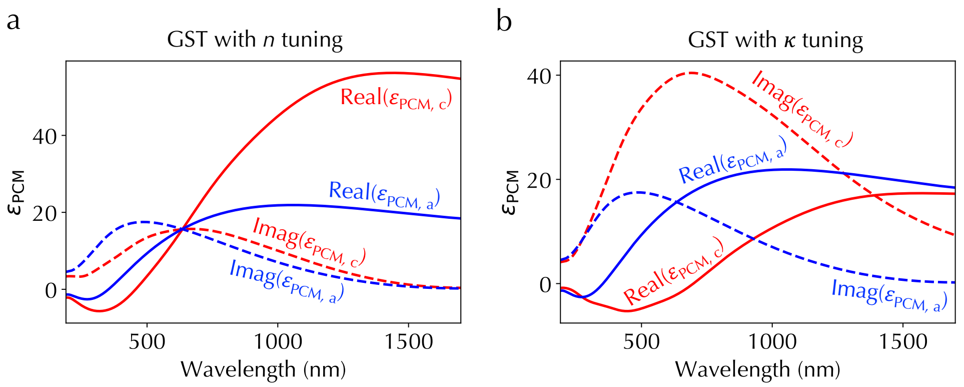

Supplementary Fig. 14 Complex-valued dielectric functions for artificial GST films with only (a) the real part (n) or (b) the imaginary part (κ) of complex-valued refractive index tuning. The red (blue) solid line is the real part of the dielectric function under the GST crystalline (amorphous) phase. The dashed red and blue lines are for imaginary parts.

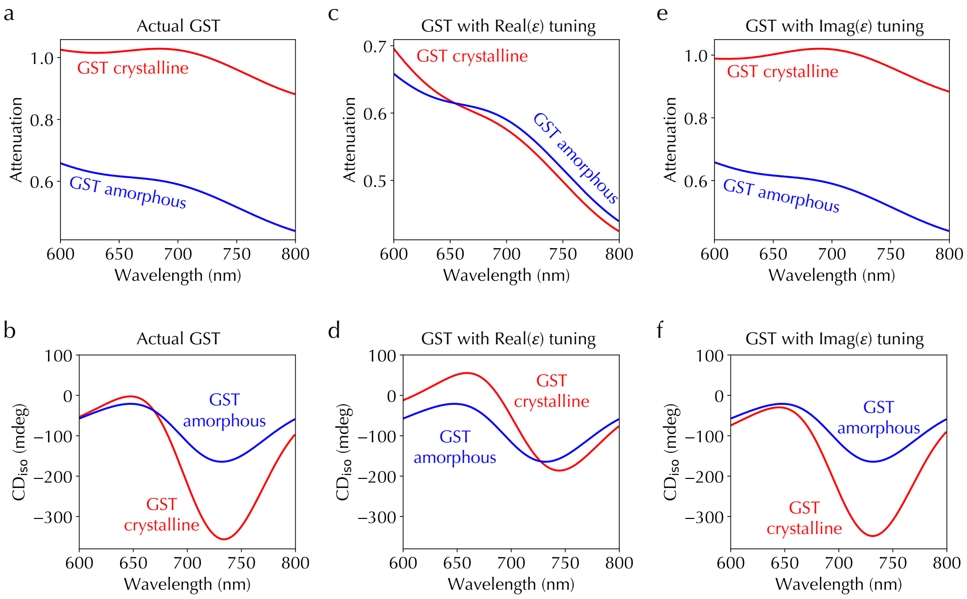

Supplementary Fig. 15 Simulation (a) absorption and (b) CD_{iso} spectra with the actual GST film. Simulation (c) absorption and (d) CD_{iso} spectra with an artificial GST film having only the real part of the dielectric function tuning. Simulation (e) absorption and (f) CD_{iso} spectra with an artificial GST film having only the imaginary part of the dielectric function tuning. Red (blue) lines are spectra under the GST crystalline (amorphous) phase. Source data are provided as a Source Data file.

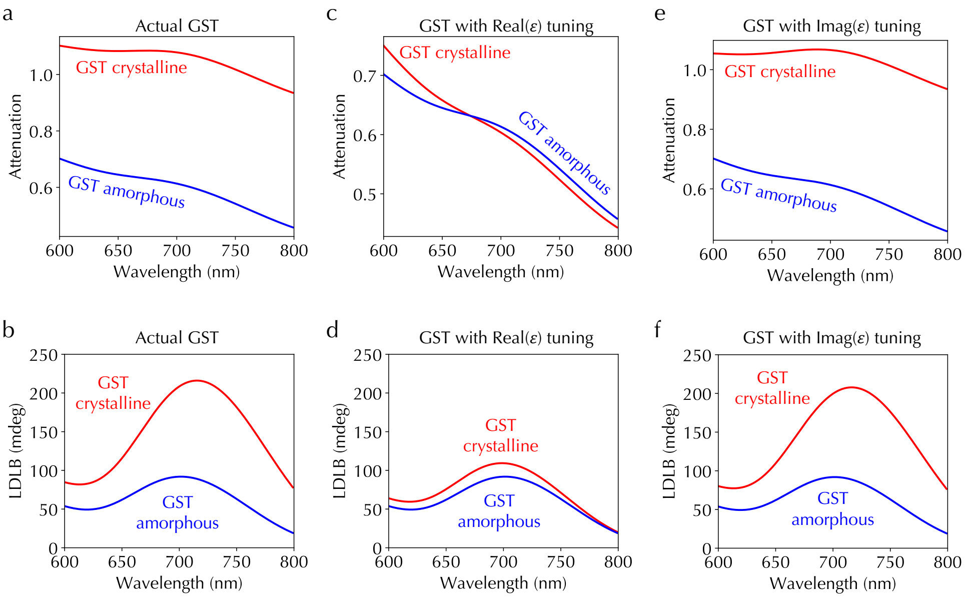

Supplementary Fig. 16 Simulation (a) absorption and (b) LDLB spectra with the actual GST film. Simulation (c) absorption and (d) LDLB spectra with an artificial GST film having only the real part of the dielectric function tuning. Simulation (e) absorption and (f) LDLB spectra with an artificial GST film having only the imaginary part of the dielectric function tuning. Red (blue) lines are spectra under the GST crystalline (amorphous) phase. Source data are provided as a Source Data file.

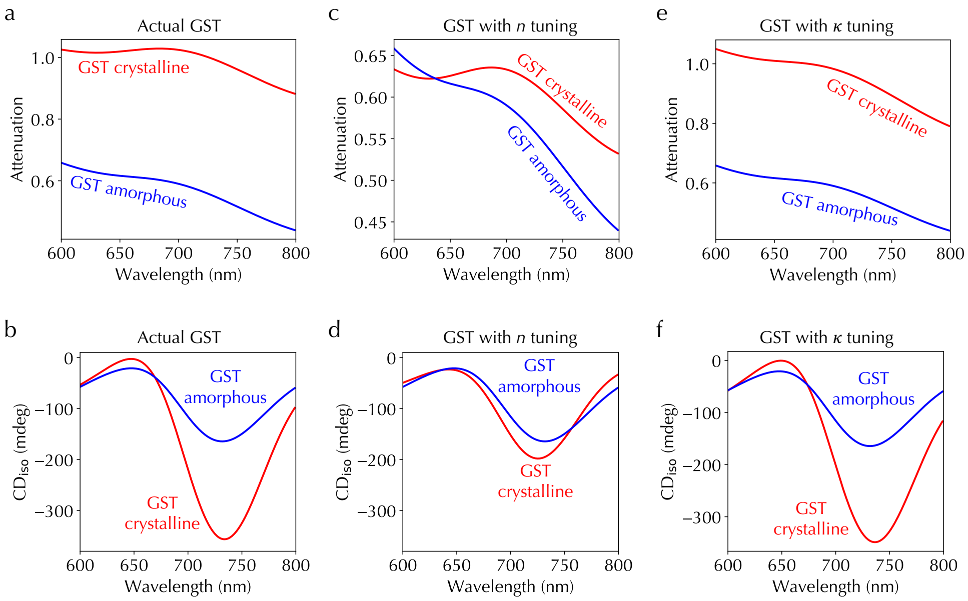

Supplementary Fig. 17 Simulation (a) absorption and (b) CD_{iso} spectra with the actual GST film. Simulation (c) absorption and (d) CD_{iso} spectra with an artificial GST film having only the real part of the refractive index tuning. Simulation (e) absorption and (f) CD_{iso} spectra with an artificial GST film having only the imaginary part of the refractive index tuning. Red (blue) lines are spectra under the GST crystalline (amorphous) phase. Source data are provided as a Source Data file.

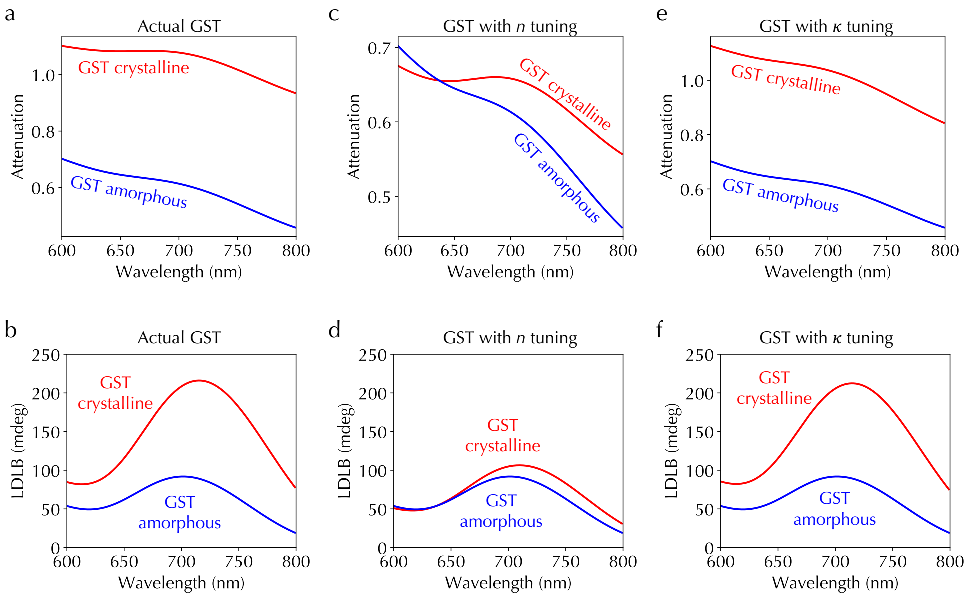

Supplementary Fig. 18 Simulation (a) absorption and (b) LDLB spectra with the actual GST film. Simulation (c) absorption and (d) LDLB spectra with an artificial GST film having only the real part of the refractive index tuning. Simulation (e) absorption and (f) LDLB spectra with an artificial GST film having only the imaginary part of the refractive index tuning. Red (blue) lines are spectra under the GST crystalline (amorphous) phase. Source data are provided as a Source Data file.

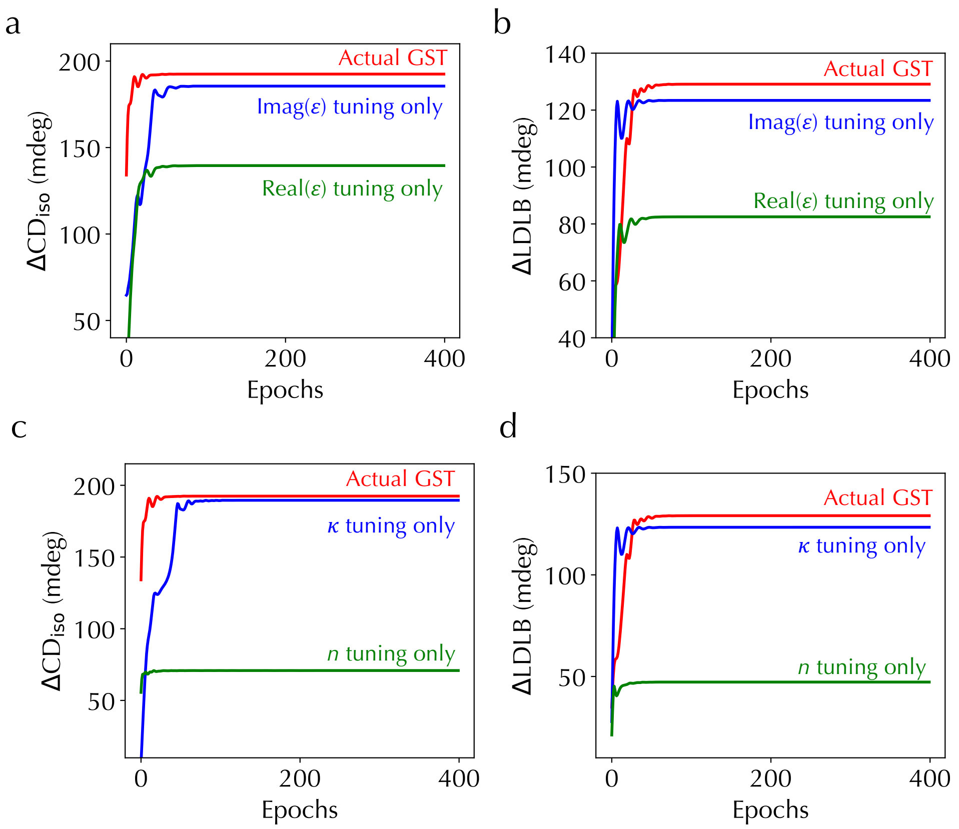

Supplementary Fig. 19 Training curves of (a) ΔCD_{iso} and (b) ΔLDLB for the actual GST film and artificial GST films having only the real or imaginary parts of the dielectric function tuning, respectively. (c), (d) Corresponding training curves for the actual GST film and artificial GST films having only the real or imaginary parts of the refractive index tuning, respectively. The red lines are for the actual GST film. The blue (green) lines are for artificial GST films with only real (imaginary) parts tuning. Source data are provided as a Source Data file.

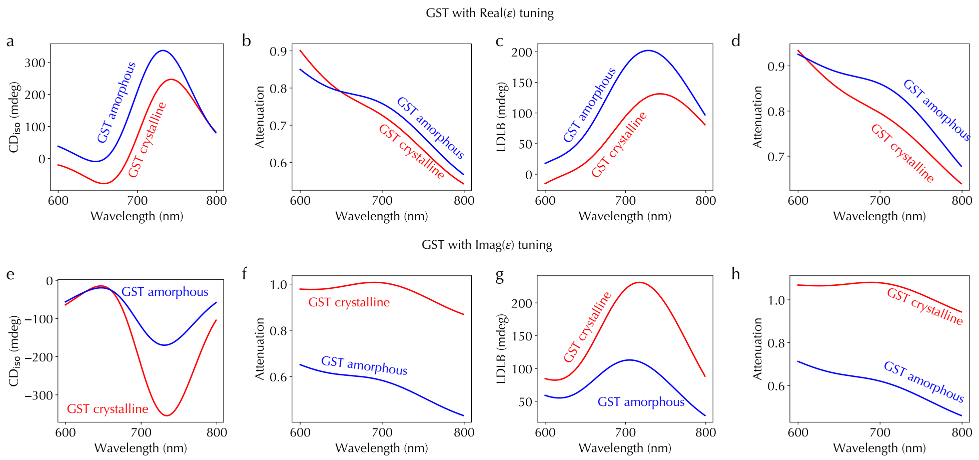

Supplementary Fig. 20 Simulation (a) CD_{iso}, (b) corresponding absorption, (c) LDLB, and (d) corresponding absorption spectra of the optimized heterostructure with the artificial GST film having only the real part of the dielectric function tuning, respectively. Simulation (e) CD_{iso}, (f) corresponding absorption, (g) LDLB, and (h) corresponding absorption spectra of the optimized heterostructure with the artificial GST film having only the imaginary part of the dielectric function tuning, respectively. Source data are provided as a Source Data file.

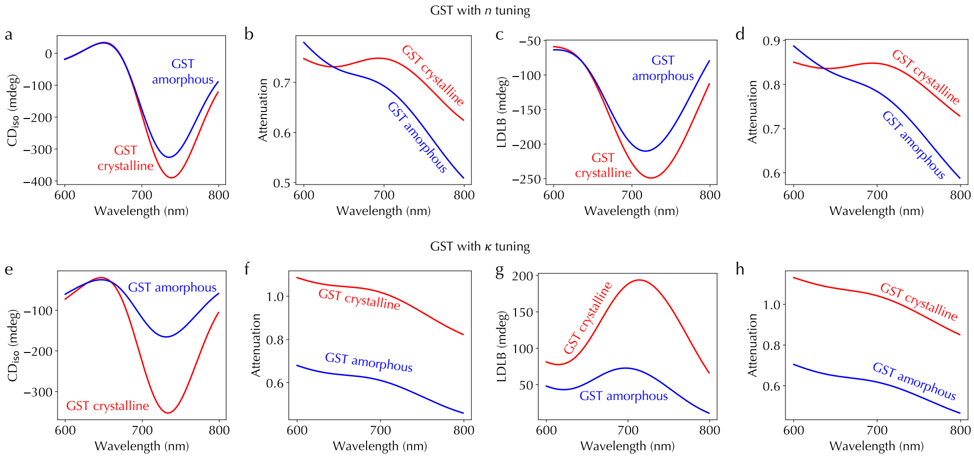

Supplementary Fig. 21 Simulation (a) CD_{iso}, (b) corresponding absorption, (c) LDLB, and (d) corresponding absorption spectra of the optimized heterostructure with the artificial GST film having only the real part of the refractive index tuning, respectively. Simulation (e) CD_{iso}, (f) corresponding absorption, (g) LDLB, and (h) corresponding absorption spectra of the optimized heterostructure with the artificial GST film having only the imaginary part of the refractive index tuning, respectively. Source data are provided as a Source Data file.

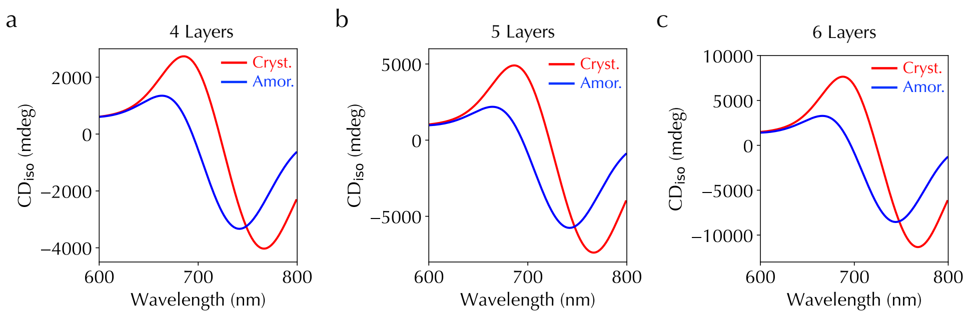

Supplementary Fig. 22 Simulation CD_{iso} spectra of optimized heterostructures containing (a) four, (b) five, and (c) six layers of twisted aligned CNT films under GST crystalline (red lines) and amorphous (blue lines) phases. Source data are provided as a Source Data file.

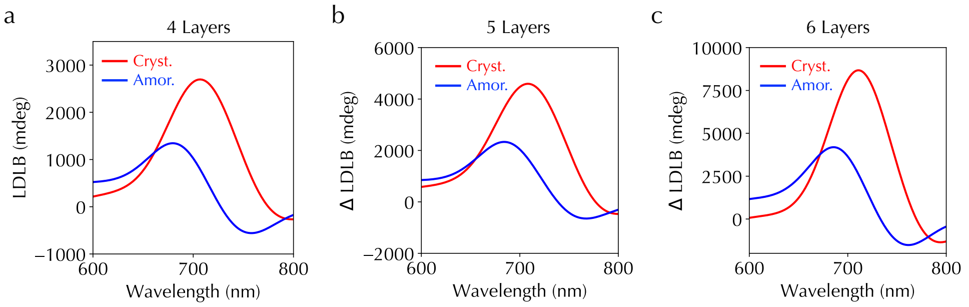

Supplementary Fig. 23 Simulation LDLB spectra of optimized heterostructures containing (a) four, (b) five, and (c) six layers of twisted aligned CNT films under GST crystalline (red lines) and amorphous (blue lines) phases. Source data are provided as a Source Data file.

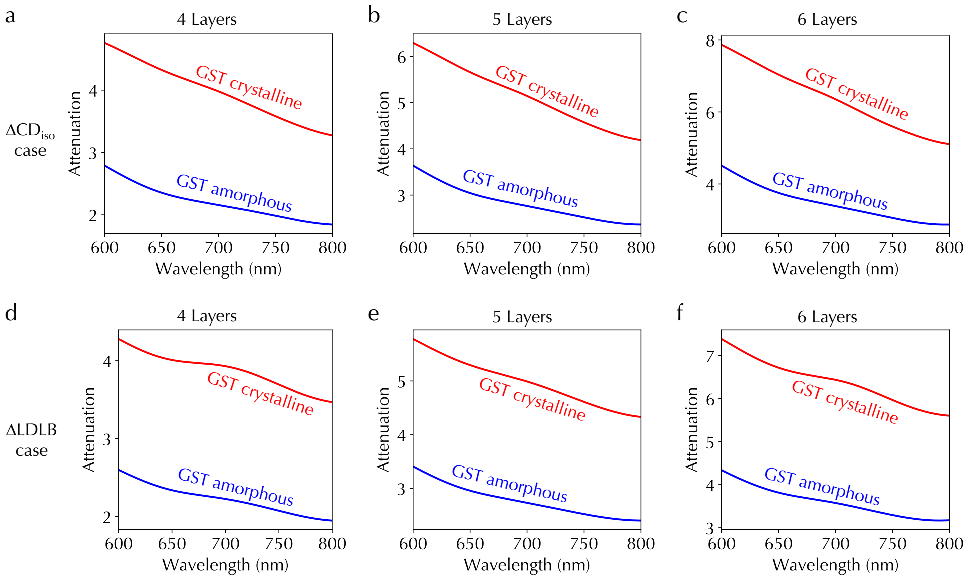

Supplementary Fig. 24 Simulation absorption spectra of optimized heterostructures containing (a) four, (b) five, and (c) six layers of twisted aligned CNT films, corresponding to Fig. 4c of the main text, under GST crystalline (red lines) and amorphous (blue lines) phases. (d)–(f) Corresponding spectra of optimized heterostructures in Fig. 4d of the main text. Source data are provided as a Source Data file.

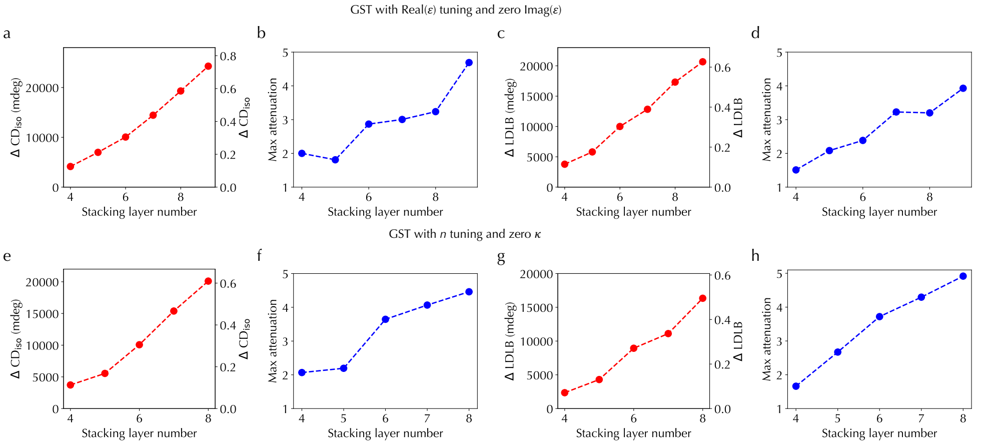

Supplementary Fig. 25 (a) $\Delta\mathrm{CD_{iso}}$ and (b) corresponding maximum attenuation, and (c) ΔLDLB and (d) corresponding maximum attenuation as a function of the number of stacked layers of twisted aligned CNT films, with the artificial GST film with the tunable real part of the dielectric function and zero imaginary part, respectively. (e) $\Delta\mathrm{CD_{iso}}$ and (f) corresponding maximum attenuation, and (g) ΔLDLB and (h) corresponding maximum attenuation as a function of the number of stacked layers of twisted aligned CNT films, with the artificial GST film with the tunable real part of the refractive index and zero imaginary part, respectively. Source data are provided as a Source Data file.

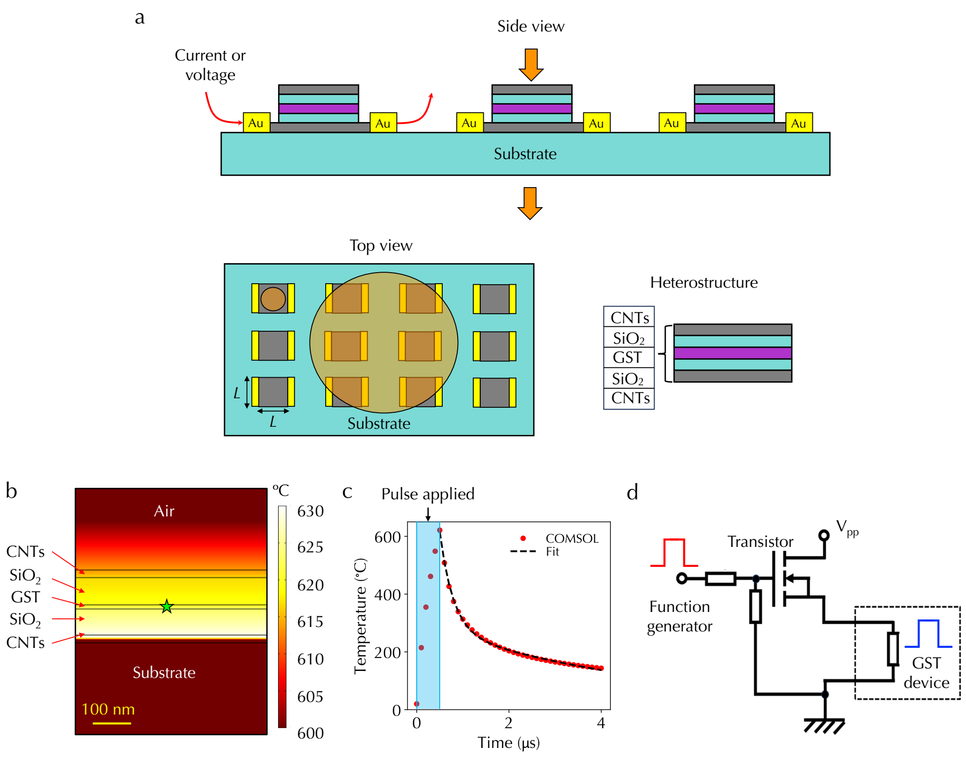

Supplementary Fig. 26 (a) Illustration of a spatial-light-modulator-type device containing an array of chiroptical heterostructures with a small footprint. Each unit can be individually electrically reprogrammable and the circularly polarized light can either separately interact with each unit or collectively interact with the whole array. (b) Spatial temperature distribution at the time of the pulse end. (c) Time-dependent temperature (red dots) at the center of the GST film (green star in (b)) and double exponential function fitting (black dashed line). (d) Diagram of an example electrical driving circuit for controlling phase transitions in GST films. Source data are provided as a Source Data file.

a

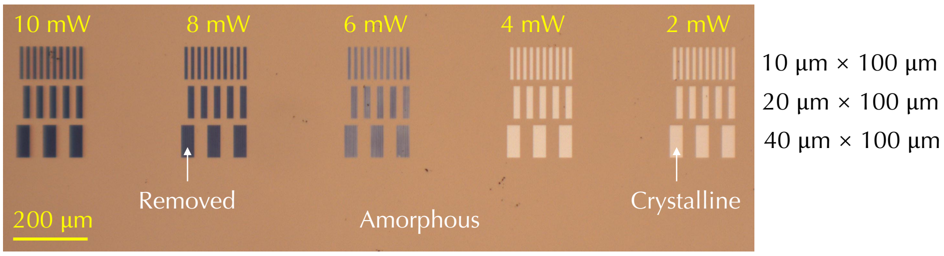

b

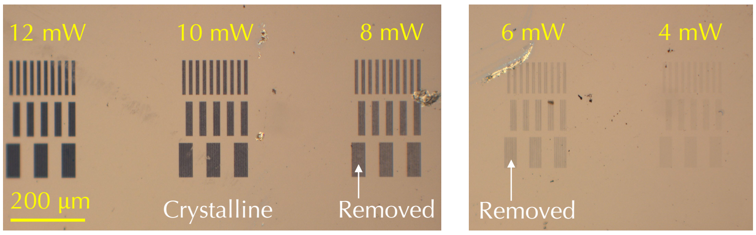

Supplementary Fig. 27 An optical microscope photo of a series of direct-laser-written rectangular patterns with dimensions of 10 μm × 100 μm, 20 μm × 100 μm, and 40 μm × 100 μm on (a) an amorphous GST film and (b) a crystalline GST film under various laser powers.
