# Supplementary material for: A Programmable Wafer-scale Chiroptical Heterostructure of Twisted Aligned Carbon Nanotubes and Phase Change Materials
Source: Nat Commun. 2025 May 14;16:4478. doi: 10.1038/s41467-025-59600-w (PMC12078695; doi:10.1038/s41467-025-59600-w)
Supplement: Supplementary file 2 — Description of Additional Supplementary Information [file 41467_2025_59600_MOESM2_ESM.docx]

**Description of Additional Supplementary Files:**

**Supplementary Movie 1:** Phase change in a chiroptical heterostructure
